# Supplementary material for: Qualitative study evaluating the expectations and experiences of Dutch parents of children with chronic gastrointestinal symptoms visiting their general practitioner
Source: BMJ Open. 2023 May 16;13(5):e069429. doi: 10.1136/bmjopen-2022-069429 (PMC10193100; doi:10.1136/bmjopen-2022-069429)
Supplement: Supplementary data [file bmjopen-2022-069429supp001.pdf]

Supplementary File 1. Interview guide [a]

I. Introduction

- Formal introduction and explantation about the interview, including the following:
  - Study aim, "to evaluate the expectations and experiences of children with chronic gastrointestinal symptoms and their parents when visiting their GP." Explain that our goal is to improve the care provided to these children.
  - That interviews will be audio- and video-recorded for processing and analysis.
  - That all respondents will be offered a final check to indicate if they agree.
- Ask if they have any last questions or remarks before recording starts.

START RECORDING

- Explain the following:
  - That shared information will be handled confidentially and anonymously and will not be shared with the relevant GP.
  - Duration: maximum of 1 hour.
  - A synopsis of interview will be sent for a member check after the interview.

II. Opening question

Could you tell me something about your/your child's gastrointestinal symptoms?

III. Topics

1. Incentives

Why did you decide to visit your GP for the gastrointestinal symptoms?

- Expansion questions:
  - Were you concerned about the symptoms? If so, what was your biggest concern? (Serious illness / impact on daily life / school absenteeism / child's suffering.)
  - What are your views about the cause of the symptoms (serious illness / stress related / innocent)?
  - How would you describe your relationship with your GP?

2. Expectations

I would like you to go back to the day you visited the GP for the first time for the gastrointestinal symptoms. What did you hope what was going to happen?

- Possible expectations:
  - Sympathetic ear.
  - Rule out physical illness (further medical investigations).
  - Find an explanation for the symptoms.

[a] This guide has been altered slightly to make it comprehensible outside the research group

  - Symptom relief (e.g. a change in eating pattern or medication).
  - Advice on how to deal with the symptoms (whether to go to school / whether to give the child attention).

3. Experiences

How did you feel when you left the GP practice?

- Suggested feelings:
  - Taken seriously / not understood / like I waisted the GP's time / relieved / concerned/ anxious
- Expansion questions
  - What made you feel like that?
  - How did the GP contribute to this feeling?

Did the GP fulfil your expectations?

How would you score the GP's performance in the consultation on a scale from 1 to 10?

- Expansion questions: (e.g., if the answer is 7/10) what should the GP have done to turn the 7 into an 8?
- Possible improvements: Further medical investigations / referral / treatment / listen better / better explanation about the cause of the symptoms.

If you could ask the GP a question about the gastrointestinal symptoms right now, what would it be?

- Notes: Diagnosis / further steps in treatment / guidance over time

Do you now hold different views about the symptoms compared to before the GP consultation?

4. Explanation for the symptoms

What explanation did the GP provide for the symptoms? Did this explanation help you?

IV. Finishing question

Before I end the recording: is there anything else you would like to share with me?

END RECORDING

V. Important questions for all themes:

If I would ask your parents the same question, what would they answer?

If I would ask your son/daughter the same question, what would he/she answer?

[a] This guide has been altered slightly to make it comprehensible outside the research group
