# Supplementary material for: Qualitative study evaluating the expectations and experiences of Dutch parents of children with chronic gastrointestinal symptoms visiting their general practitioner
Source: BMJ Open. 2023 May 16;13(5):e069429. doi: 10.1136/bmjopen-2022-069429 (PMC10193100; doi:10.1136/bmjopen-2022-069429)
Supplement: Supplementary data [file bmjopen-2022-069429supp002.pdf]

Supplementary File 2. Responses to the member check

Member check 1: Synopsis of the interview – All 15 respondents

Four respondents informed us that they agreed with the synopsis.

Ten respondents did not respond to the synopsis.

One mother made amendments to the synopsis clarifying her daughter's specific symptoms, why she had decided to visit the GP and how she and her husband deal with problems concerning the health of their children.

Member check 2. Conceptual framework exemplified by the interview – 3 respondents

One respondent informed us that they agreed with how their story was represented in the conceptual framework.

One respondent did not respond to our e-mail.

One respondent let us know the following via e-mail:

> *The way you have outlined the story is correct for 90%. The last part, about the relationship between me and the GP, is not completely right. When I went to the doctor for my daughter, we were seen by a representative of the GP practice. So not my own doctor. I assume her work is monitored, since my own GP called me to ask if I wanted to participate in the trial. This was in response to my visit. So, he did receive the signal. However, it is true that he also did not perform further investigations to find the cause of my daughter's symptoms. However, I do completely trust my own GP. I do not visit him often, but when I visit him, I feel taken seriously. For example, my other daughter has been admitted to the hospital due to the RS virus because he took her symptoms seriously. But this time it was a representative. Maybe it is true that my trust in her is not as high as in my own GP. But I thought that the last part in the text somewhat wronged my own GP. So that is my only remark. Besides that: fine!*

After making changes in the document clarifying that the GP discussed was not the mother's regular GP, she responded as follows:

> *The adjustment is very minimal. The reader could still interpret the story differently (as seen from my perspective). However, I do understand that this text is created with a certain aim. From that perspective, very well, use it like this. Thank you for discussing this with me.*
