# Supplementary material for: Qualitative study evaluating the expectations and experiences of Dutch parents of children with chronic gastrointestinal symptoms visiting their general practitioner
Source: BMJ Open. 2023 May 16;13(5):e069429. doi: 10.1136/bmjopen-2022-069429 (PMC10193100; doi:10.1136/bmjopen-2022-069429)
Supplement: Author's manuscript [file bmjopen-2022-069429.draft_revisions.pdf]

BMJ Open

BMJ Open is committed to open peer review. As part of this commitment we make the peer review history of every article we publish publicly available.

When an article is published we post the peer reviewers' comments and the authors' responses online. We also post the versions of the paper that were used during peer review. These are the versions that the peer review comments apply to.

The versions of the paper that follow are the versions that were submitted during the peer review process. They are not the versions of record or the final published versions. They should not be cited or distributed as the published version of this manuscript.

BMJ Open is an open access journal and the full, final, typeset and author-corrected version of record of the manuscript is available on our site with no access controls, subscription charges or pay-per-view fees (http://bmjopen.bmj.com).

If you have any questions on BMJ Open's open peer review process please email info.bmjopen@bmj.com

Qualitative study evaluating the expectations and experiences of parents of children with chronic gastrointestinal symptoms visiting their general practitioner

Journal:	*BMJ Open*
Manuscript ID	bmjopen-2022-069429
Article Type:	Original research
Date Submitted by the Author:	24-Oct-2022
Complete List of Authors:	Ansems, Sophie; University Medical Centre Groningen, Department of General Practice and Elderly Care Medicine<br>Ganzevoort, Ilse; University Medical Centre Groningen, General Practice and Elderly Care Medicine<br>van Tol, Donald; University Medical Centre Groningen, Department of General Practice and Elderly Care Medicine; University Medical Centre Groningen, Department of Sociology, Faculty of Behavioral and Social Sciences<br>Fokkema , Tryntsje; University Medical Centre Groningen, Department of General Practice and Elderly Care Medicine<br>Olthof, Marijke; University Medical Centre Groningen, Department of General Practice and Elderly Care Medicine<br>Berger, Marjolein; University Medical Centre Groningen, Department of General Practice and Elderly Care Medicine<br>Holtman, G.; University Medical Centre Groningen, Department of General Practice and Elderly Care Medicine
Keywords:	Functional bowel disorders < GASTROENTEROLOGY, PRIMARY CARE, QUALITATIVE RESEARCH

SCHOLARONE™
Manuscripts

I, the Submitting Author has the right to grant and does grant on behalf of all authors of the Work (as defined in the below author licence), an exclusive licence and/or a non-exclusive licence for contributions from authors who are: i) UK Crown employees; ii) where BMJ has agreed a CC-BY licence shall apply, and/or iii) in accordance with the terms applicable for US Federal Government officers or employees acting as part of their official duties; on a worldwide, perpetual, irrevocable, royalty-free basis to BMJ Publishing Group Ltd ("BMJ") its licensees and where the relevant Journal is co-owned by BMJ to the co-owners of the Journal, to publish the Work in this journal and any other BMJ products and to exploit all rights, as set out in our licence.

The Submitting Author accepts and understands that any supply made under these terms is made by BMJ to the Submitting Author unless you are acting as an employee on behalf of your employer or a postgraduate student of an affiliated institution which is paying any applicable article publishing charge ("APC") for Open Access articles. Where the Submitting Author wishes to make the Work available on an Open Access basis (and intends to pay the relevant APC), the terms of reuse of such Open Access shall be governed by a Creative Commons licence – details of these licences and which Creative Commons licence will apply to this Work are set out in our licence referred to above.

Other than as permitted in any relevant BMJ Author's Self Archiving Policies, I confirm this Work has not been accepted for publication elsewhere, is not being considered for publication elsewhere and does not duplicate material already published. I confirm all authors consent to publication of this Work and authorise the granting of this licence.

For peer review only

Qualitative study evaluating the expectations and experiences of parents of children with chronic gastrointestinal symptoms visiting their general practitioner

Sophie M. Ansems [1], MD, PhD candidate | ORCID 0000000287448224 | s.m.ansems@umcg.nl

Ilse N. Ganzevoort [1], MSc, PhD candidate | i.n.ganzevoort@umcg.nl

Donald G. van Tol [1,2], PhD, Assistant Professor. | d.g.van.tol@umcg.nl

Tryntsje Fokkema [1], PhD, Researcher | tryntsjefokkema@gmail.com

Marijke Olthof [1], MD PhD, GP and researcher | marijkeolthof@gmail.com

Marjolein Y. Berger [1], MD PhD, Professor | m.y.berger@umcg.nl

Gea A. Holtman [1], PhD, Assistant Professor |ORCID 000000016579767X | g.a.holtman@umcg.nl

[1] Department of General Practice, University Medical Center Groningen, University of Groningen, the Netherlands

[2] Department of Sociology, Faculty of Behavioral and Social Sciences, University of Groningen, the Netherlands

Corresponding author: Sophie M. Ansems | Department of General Practice and Elderly Care Medicine, FA21, University of Groningen, University Medical Centre Groningen, PO Box 196, 9700 AD Groningen, the Netherlands | E-mail: s.m.ansems@umcg.nl | +31 6 14186134

Word count: 4099

Key words: functional gastrointestinal disorders, primary health care, children's health, general practitioners, qualitative research

For peer review only

ABSTRACT

Objectives: Chronic gastrointestinal symptoms are common among children and affect their daily activities and quality of life. The majority will be diagnosed with a functional gastrointestinal disorder. Effective reassurance and education are therefore key components of the physician's management. Qualitative studies have shown how parents and children experience specialist paediatric care, yet less is known about GPs, who manage most cases in the Netherlands and have a more personal and enduring relationship with their patients. Therefore, this study evaluates the expectations and experiences of parents of children visiting a GP for chronic gastrointestinal symptoms.

Design: We conducted a qualitative interview study. Online interviews were audio and video recorded, transcribed verbatim and independently analysed by the first 2 authors. Data were collected and analysed concurrently until data saturation was reached. Using thematic content analysis, we developed a conceptual framework reflecting respondent expectations and experiences. We performed a member check of the interview synopsis and the conceptual framework.

Setting: Dutch primary care.

Participants: We purposively sampled participants from a randomized controlled trial evaluating the effectiveness of faecal calprotectin testing in children with chronic gastrointestinal complaints in primary care. Thirteen parents and 2 children participated.

Results: Three key themes emerged: disease burden, GP–patient relationship, and reassurance. Often, the experienced disease burden and the pre-existing GP–patient relationship influenced expectations (e.g., for further investigations or a sympathetic ear), and when a GP fulfilled these expectations, a trusting GP–patient relationship ensued that facilitated reassurance. We found that individual needs influenced these themes and their interrelationships.

Conclusion: Insights provided by this framework could help GPs managing children with chronic gastrointestinal symptoms in daily practice and may therewith improve the consultation experience for parents. Further research should evaluate whether this framework also holds true

For peer review only - http://bmjopen.bmj.com/site/about/guidelines.xhtml

for children.

STRENGTHS AND LIMITATIONS OF THIS STUDY

- This is the first study to specifically address parental expectations of and experiences with a GP visit for paediatric chronic gastrointestinal symptoms.
- Coding and analysis was done by several researchers and two separate member checks were performed, increasing the study's credibility.
- Although our intention was to study the expectations and experiences of children as well, only 2 children participated in this study.
- Despite purposive sampling, all participants had a typical Dutch cultural background.

For peer review only - http://bmjopen.bmj.com/site/about/guidelines.xhtml

ABBREVIATIONS

COREQ	Consolidated Criteria for Reporting Qualitative Research
FGID	Functional gastrointestinal disorders
FSS	Functional somatic symptoms
GP	General Practitioner
RCT	Randomised controlled trial
SRQR	Standard Reporting of Qualitative Research

For peer review only

INTRODUCTION

A Dutch general practitioner (GP) will typically see approximately 10 children with chronic gastrointestinal (GI) symptoms each year (1,2), of whom about 9 will have a functional gastrointestinal disorder (FGID) (2,3). FGID (e.g. functional constipation) is one of the symptom clusters of 'functional somatic symptoms' (FSS, i.e. symptoms that cannot be fully explained by well-defined psychiatric or somatic illness) (3,4). Other FSS clusters in children are pain, fatigue and cardiopulmonary symptoms (4). In these settings, GPs can reassure and educate parents and children through various strategies, including normalisation (5), explanation (6,7), affective reassurance (8), the exclusion of physical illness (6,9), and the provision of reassurance through normal test results (10–12). However, research has shown that 50% of children with FGID still have abdominal pain that affects daily activities 12 months after first presenting (13), while other research has shown an association between FGID and the child's quality of life, school absenteeism, anxiety, and depression (14,15).

Most current reassurance and education strategies are planned from the GP's perspective, with evidence that GPs do not always assess patient expectations and needs adequately (12). Qualitative studies among children and adolescents with FGID revealed their desire of a clear diagnosis provided by their treating physician (16–18). However, qualitative studies performed in paediatric specialist care have shown that pre-existing concerns (e.g. about serious illness or the impact of symptoms) and expectations of the doctor (e.g. to exclude physical illness or help manage pain) varied considerably among parents (19–22), creating uncertainty about the best reassurance strategy (23). Parents may also hold different views about the treating doctor, perhaps believing another professional would be better suited to treat their child or that the doctor does not consider psychologically based symptoms to be genuine (19). Other parents report that they want guidance and follow-up by their usual GP or medical professional (21). Indeed, most children with chronic GI symptoms are managed in primary care in the Netherlands (2), yet most research has taken place in paediatric specialist care (16–22). We hypothesised that the more personal and enduring relationship between GPs and patients could influence how

For peer review only - http://bmjopen.bmj.com/site/about/guidelines.xhtml

parents and children experience consultations. In this study, we aimed to evaluate the expectations and experiences of parents and children when visiting a GP with chronic GI symptoms through a qualitative interview study.

For peer review only

For peer review only - http://bmjopen.bmj.com/site/about/guidelines.xhtml

METHODS

Study design

We used an inductive-deductive methodology (24), meaning we used both pre-existing insights and literature as well as new insights that arose during the analysis of our data. We included parents and children living in the northern part of the Netherlands and collected data between January and June 2021. To allow personal and sensitive themes to arise, we collected data via in-depth semi-structured interviews (25). We used the Consolidated Criteria for Reporting Qualitative Research (COREQ) checklist (25) and the Standard Reporting of Qualitative Research (SRQR) checklist (25,26). This study was approved by the Medical Research Ethics Committee of the University Medical Center Groningen (Research Register: 202000795).

Respondents

Parents and children engaged in an ongoing randomised controlled trial (RCT) evaluating the clinical and cost-effectiveness of faecal calprotectin testing in primary care were eligible to take part (27). The trial included children aged 4–18 years who visited their GP with chronic abdominal pain and/or chronic diarrhoea and excluded children with a history of chronic organic GI disease or who had received endoscopic evaluation, paediatric referral for GI symptoms, or faecal calprotectin testing within the preceding 6 months. The first author approached parents and/or children by telephone after receiving informed consent for their participation in the RCT and related research.

For the present study, we used purposive sampling by age, sex (children and parents), parental educational level, and parental country of birth. Inclusion continued until data saturation, defined as being when interviews generated no new information on the research questions. All respondents received an e-mail with a patient information letter and provided digital informed consent, which consistent with Dutch law, was obtained either from parents (child age <12 years), parents and child (age 12–15 years) or the child only (age >15 years).

Data collection

An interview guide was prepared using sensitising concepts from the literature and expert

For peer review only - http://bmjopen.bmj.com/site/about/guidelines.xhtml

discussion. Sensitising concepts comprised reassurance and education strategies, parental worry cognitions, parental and child expectations, views about the GP, and experiences of the GP consultation (5,6,22,23,7,8,10–12,19–21). The interview guide contained open-ended questions concerning the incentives, expectations, and experiences related to the visit, as well as the GP's explanation for the symptoms (Supplementary File 1).

The first author, a female physician and researcher trained to perform qualitative interviews, conducted the semi-structured interviews. In the interview setting, she presented herself as a researcher and not a physician. By doing so, she prevented confusion about her role and gave the respondents the feeling they could speak freely about their GP. Another primary care researcher who is not a physician (second author, a PhD candidate studying FGID in children) observed all interviews. Before starting, we emphasised the confidential nature of the interview, that only anonymised transcripts would be kept, deleting any recordings, and that data would not be discussed with their GP. Children aged ≥12 years could decide if a parent accompanied them, but we only interviewed the parents for children aged <12 years. Interviews were held online (via Microsoft Teams) due to the COVID-19-pandemic and at a time convenient for respondents. They lasted 15–45 minutes, were audio and video recorded, and transcribed verbatim by the interviewer. Quotes were translated from Dutch to English by the first author and subsequently edited by a native English speaker and editor. Finally, the first author checked whether their meaning retained. Parents and/or children received an interview synopsis to perform a member check.

Analysis

Data were collected and analysed concurrently, allowing emerging themes to be incorporated and explored in subsequent interviews. Thematic content analysis was conducted as proposed by Braun and Clarke (28). The first and second authors coded the first three transcripts separately for standardisation. The first author then analysed the remaining transcripts and the second author independently checked the coding results before they discussed and resolved inconsistencies by consensus. Finally, two other members of the research team (a sociologist and

For peer review only - http://bmjopen.bmj.com/site/about/guidelines.xhtml

a primary care researcher) checked the coding tree. Any remaining inconsistencies and missing codes were discussed during a research team meeting.

Throughout the analysis, memos were written, including notes about the identified themes and their relationships, and provisional findings were critically discussed in research team meetings. We developed an initial conceptual framework after four meetings, following which the first author checked and confirmed whether it could be applied to each respondent's story. Further member checking was undertaken by inviting three respondents to comment on their own story. All analyses were facilitated by the use of Atlas.ti 9.1 software.

Patient and public involvement

Parents and children were involved in the interviews and its interpretation. Additionally, the results were disseminated among them, as will the manuscript once published.

For peer review only

For peer review only - http://bmjopen.bmj.com/site/about/guidelines.xhtml

RESULTS

Respondents

We approached 23 eligible participants by telephone, of which 18 agreed to receive further information. Two parents and one child declined to participate either for unknown reasons, personal circumstances, or not wanting to talk (1 each). Data saturation first appeared after ten interviews when we had only interviewed 1 child aged ≥12 years and 1 father. Therefore, we continued purposive recruitment of these groups for an additional five interviews to improve representation and saturation. Unfortunately, only 1 more child was recruited. All interviews took place within 2 months after the initial presentation. Table 1 shows the characteristics of the 15 respondents. The results of the initial member check for these 15 respondents are shown in Supplementary File 2 (member check 1), detailing that only one mother returned the synopsis with clarifying information.

Themes

We discovered three themes concerning the expectations and experiences of parents when visiting a GP for chronic GI symptoms: disease burden, GP–patient relationship, and reassurance, with specific needs affecting their interrelationship. Using illustrative quotations, we discuss each theme's meaning, subthemes (Table 2), and relation to the expectations and experiences of respondents, seeking to reveal the relationship between themes reinforced by respondent need (conceptual framework in Figure 1). Three cases were purposively selected for additional member check (Supplementary File 2, member check 2), as detailed in Figure 2A-2C as representative examples.

For peer review only - http://bmjopen.bmj.com/site/about/guidelines.xhtml

Table 1. Parents' and children's characteristics (*n*=15)

Characteristic	Number
Gender child, male	7
Age child, years	
4 -7	5
8 - 11	8
12 - 15	1
16 - 17	1
Urban area of residence	5
Country of birth of both parents	
The Netherlands	15
Educational level mother [a]	
Low	1
Medium	6
High	8
Educational level father [a]	
Low	2
Medium	6
High	7
Paid employment	
Both parents	14
One parent	1
Marital status parents	
Married / living together	11
Divorced	4
Present at interview	
Child	1
Child and mother	1
Mother	8
Father	2
Both parents	3
Diagnosis after 6 months	
Functional constipation	9
Irritable bowel syndrome	2
Functional constipation and functional abdominal pain	2
Functional abdominal pain	2

[a] Educational level was divided in low (lower secondary education or lower), intermediate (secondary (vocational) education) and high (Bachelor's degree or higher) (29,30).

For peer review only

Table 2. Subthemes per theme and factors positively and negatively influencing each theme

	Subtheme	*Positive influence*	*Negative influence*
Disease burden	**Duration**	Mild symptoms	Long duration of symptoms
		Stable symptoms	Recurring symptoms
		Short symptom duration	
	**Additional symptoms Impact**		Additional symptoms (e.g. weight loss)
		No limitations due to symptoms	Symptoms impact rest of the family
		-	Child is limited in everyday life due to symptoms (e.g. school absenteeism, sports)
	**Concerns**	No concerns about symptoms	Concerns about symptoms
		No concerns because good explanation for symptoms (e.g. constipation, or runs in family)	Concerns about additional symptoms (e.g. weight loss)
		-	Concerns about impact on everyday life
	**History**	-	Parent recognise symptoms from their own medical history
		-	Other health care providers could not relieve the symptoms (e.g. psychotherapist, alternative medicine)
Doctor–patient relationship	**Pre-existing relationship**	Long-lasting doctor–patient relationship	First time at this GP
		Positive previous experience with GP	Negative previous experience with GP for chronic abdominal symptoms
	**Consultation**	Parents felt heard and understood	Not enough room in consultation for respondent's story
		GP focused on and comforted child	Short duration of consultation
		GP showed personal interest	GP did not answer main request
		GP fulfilled expectations	GP normalised or downplayed symptoms
		GP and respondents together decided treatment plan	GP showed no empathy
	**Follow-up**	GP planned follow-up consultation	No planned follow-up consultation
		Low threshold to make new GP appointment	Parent had to contact GP about continuation
		-	GP does not take enough initiative
		-	GP provided no help besides medical care (e.g. psychosocial care)
		-	GP did not provide a solution
Cognitive reassurance	**Explanation**	Matching explanatory model	Non-matching explanatory model
		Explanatory model fulfilled the respondent's expectation (e.g. constipation)	GP provided unclear or no explanatory model
		Respondents and GP together explored different explanatory models	GP did not seem to believe in explanatory model
		-	Respondents were unsure about explanation
	**Physical interventions**	Successful treatment (e.g. Forlax® relieved symptoms)	Unsuccessful treatment
		Physical examination confirmed diagnosis	Medical tests did not reveal an explanation
			Parents wished further medical investigation
		-	GP provided 'quick fix' for long-lasting problem (e.g. medication)

For peer review only - http://bmjopen.bmj.com/site/about/guidelines.xhtml

Disease burden

The disease burden experienced by parents influenced the expectations of GPs. Burden comprised the duration and severity of symptoms, the presence of additional symptoms (e.g., weight loss), the impact on everyday life, and the degree of concern about the symptoms (Table 2). Some parents experienced low disease burden (e.g., when in an early stage of symptoms), while others experienced chronic recurring GI symptoms that had limited everyday life for years.

> Mother (M8): *For one year he was pain free and… during the last couple of months it returned. He has lost… weight, and the pain is limiting him in daily life… and in school. He arrived late to school because his belly hurt and he had to go to the toilet again. So, I just wanted further investigation.*

Most parents with a low disease burden emphasised that they had no specific expectations of the GP.

> Mother (M6): *Yes, exactly* [the GP fulfilled my expectations]. *Because it was not a severe situation, I did not have high expectations. I did not have a tense feeling,… so… GPs can help just by being friendly.*

Parents had higher expectations with higher disease burdens, typically expressing concern that 'something must be wrong'. This often resulted in questions about the cause of symptoms and expectations for the GP to label symptoms, conduct further investigations, or refer to secondary care.

> Father (F1): *At some point you are fed up… you want to know the answer, where it could come from? I know that my symptoms are clearly due to stress and everything, but I do not think that is the case for* [my daughter].

> Mother (M3): *And then I said 'You did not find anything but I would like it to be further investigated because 1) she is losing weight and 2) she just has an awful lot of pain attacks while turning pale, feeling bad, and not eating'. Her stool is almost never normal, so that cannot be right. But then, I got the response 'What investigations were you thinking of?'… I*

For peer review only - http://bmjopen.bmj.com/site/about/guidelines.xhtml

am no doctor! So… I said... 'Just send me to a paediatrician'… 'There must be a cause for this, this cannot be right'.

Concern about symptoms varied among the parents. A couple of parents expressed concern over additional symptoms (e.g. fever), while others worried about the impact of symptoms on everyday life (e.g. school absenteeism and disturbed sleep) or the lack of explanation. By contrast, a few parents emphasised that they usually do not worry easily about their child's health and did not in this case.

Although both children mentioned that their symptoms affected them negatively during school and that they hoped the GP could find the cause and a solution, their parents had taken the initiative to consult the GP in both cases.

Doctor–patient relationship

The doctor–patient relationship comprised two elements: the pre-existing relationship (influencing both expectations and experiences) and the relationship during and after the consultation (influencing only experiences). Most parents reported a trusting relationship with their GP, with several having long-standing relationships and good previous experiences. One father expressed that his GP '*is not only a doctor, but above all, a good human being*'.

Parents emphasised the importance of feeling heard and understood by the GP during consultations. Additionally, most appreciated the GP comforting the child and directing his/her questions and explanations to the child.

> Mother (M4): *If something is wrong… he just really takes the time for you. Sometimes I am in the waiting room and the previous consultation is running late. But, actually, I find that a good sign because it means he takes the time for you. Also, for children, he really takes the time.*

Others felt their GP downplayed the child's symptoms.

> Mother (M8): *But I do find that he could have shown a bit more empathy and… talked with my child a bit more. Because what he is feeling… is really what he is feeling. And now he has the sense that he was pretending. But it actually is… real.*

For peer review only - http://bmjopen.bmj.com/site/about/guidelines.xhtml

Parents also felt comforted when the GP planned a follow-up consultation. This made them feel that the GP had acknowledged their concerns and the disease burden.

> Mother (M2): *She completely examined* [my child], *we had a good conversation, and she* [said] *we will see and to call in 2 weeks to discuss it again… it was not like… manage it yourself and goodbye. Not at all.*

Similarly, parents felt isolated when the GP did not plan a follow-up consultation.

> Mother (M1): *If everything is ruled out* [and] *there are still symptoms, as a parent you want to do something… I would have appreciated a follow-up appointment, preferably already scheduled, so you can talk about it to find a good solution* together*. Although he cannot do anything with the test result* [everything is ruled out]*… let's keep talking…* like*... what can I help you with? Do you actually need help, or do you think you can handle it on your own?*

When younger, the 17-year-old respondent mentioned that he found his GP intimidating. However, recent consultations for his chronic GI symptoms had changed this because the GP was considered '*friendly and neat*' towards him.

Reassurance

Parents expressed to feel reassured via two different pathways; cognitive and affective reassurance. Cognitive reassurance was achieved when the GP responded to or changed their beliefs about the symptoms (their cognitions). Whether the GP provided an explanatory model for the symptoms, and the choice of model, played prominent roles in achieving cognitive reassurance. Sometimes the GP gave an explanation that matched the parents' belief system (e.g. the GP confirmed their expectation that constipation caused the symptoms), making it relatively easy to establish cognitive reassurance. However, if their child's symptoms did not conform to the explanatory model, parents experienced insecurity about the explanation for the symptoms.

> Father (F5): *That is the weird part because you know that the laxatives are for the constipation, but if a child suddenly has a fever and lies on the couch while having a hard time*

For peer review only - http://bmjopen.bmj.com/site/about/guidelines.xhtml

breathing… and has recovered within one hour… you do not think it is constipation… but… we did not know whether to call the GP again.

How the GP conveyed the explanatory model also had a considerable impact on cognitive reassurance.

Child (Ch7): *I don't know… it did feel like a bit of an oversimplification. Like… it felt like she did not know the answer herself…* [so] *she called it irritable bowel… that is how it felt.*

This was especially the case for the two children, who both expressed that they did not understand the explanations because '*the GP talked too much*' and '*used too many difficult words*'.

Some parents emphasised that positive findings during physical examination or successful treatment made them believe in a certain explanatory model.

Mother (M4): *Yes, I think* [the GP's final diagnosis was heartburn]*, although this was not said definitively…* [but] *since we put the bed up based on their advice,… I assume that it is related to heartburn.*

Other parents expressed that they wanted the GP to perform more medical tests to find an explanatory model for the symptoms. However, none of the parents mentioned to feel reassured by a normal test result.

Not all parents wanted a specific explanatory model for the symptoms. Several stated that they just followed the GP's instructions (e.g. to take laxatives and return after 2 weeks with an update) and were comfortable that the GP had not provided a specific explanatory model early in the diagnostic process. In these cases, affective reassurance played a larger role, meaning parents felt reassured because the GP gave them the feeling there was no need to worry and they felt heard and understood by the GP.

Father (F14): *She took both questions seriously… as well as our suggestions… and she found those suggestions plausible. So, she first performed a physical examination, looked at his tummy, knocked on it and she concluded that it was unlikely a purely physical cause.*

For peer review only - http://bmjopen.bmj.com/site/about/guidelines.xhtml

Relationship between the themes and needs of parents and children

The three themes not only influenced the expectations and experiences of parents but also influenced each other (Figure 1), with this differing between interviews (see Figures 2A-2C). For example, the mother in Figure 2A suffered a high disease burden, which prompted her to seek an explanation (cognitive reassurance). In Figure 2B, the strong and long-term doctor–patient relationship made the father trust his GP when told that he should not worry about his son's symptoms (affective reassurance), without a need for an explanation for the symptoms or treatment (cognitive reassurance). By contrast, the doctor–patient relationship in Figure 2C was weak, resulting in a greater need for cognitive reassurance because the GP could not offer affective reassurance.

The relationship between the themes was also reinforced by whether the GP fulfilled or neglected the needs of parents. For example, in Figure 2B, the GP fulfilled the father's need for acknowledgement of the disease burden by planning a follow-up consultation. This, in turn, decreased the disease burden. Conversely, in Figure 2A, the GP downplayed the symptoms and disregarded the need for an explanation, increasing the disease burden and need for cognitive reassurance, respectively.

For peer review only

DISCUSSION

Summary

This qualitative interview study of the expectations and experiences when visiting a GP for chronic GI symptoms revealed three key themes: disease burden, GP–patient relationship, and reassurance. The basis of our conceptual framework is that the disease burden and pre-existing GP–patient relationship influence expectations. When a GP met parental expectations, a trusting doctor–patient relationship ensued, creating an environment that allowed affective and cognitive reassurance. Within this framework, specific needs of parents influenced the themes and their interrelationships. By recognising and meeting these needs, the GP might improve the quality of parent experiences.

For peer review only

Strengths & weaknesses

We will discuss the strengths and limitations regarding the transferability, credibility and reliability of our study (31,32). We could find no other study in which parents and children were interviewed about their expectations and experiences of a GP consultation when the child has chronic gastrointestinal symptoms. This is important because, in a primary health care system such as that in the Netherlands, the GP is the first physician to see a child with chronic GI symptoms and will typically manage most cases without referral to secondary care. Therefore, our results are transferable to other countries with strong primary health care systems, such as the United Kingdom and Canada (33). Additionally, the detailed description of our findings makes them transferable to the parents of children with other FSS (e.g. chronic fatigue) in general practice, because FSS share a similar underlying multifactorial pathophysiology (34).

We interviewed a diverse population consisting of mothers, fathers, and children who came from urban and rural areas and had varying educational levels and marital statuses. However, despite our intention to interview children, we could only recruit two children aged ≥12 years. Although their expectations and experiences fit within the three themes of our conceptual framework, they also differed from the parent perspective. More importantly, the small number of participating children does not allow us to draw conclusions about their expectations and experiences. This implies that our findings are only transferable to parental expectations and experiences. Future

For peer review only - http://bmjopen.bmj.com/site/about/guidelines.xhtml

studies should elaborate on the point of view of children and evaluate whether this framework also holds true for their expectations and experiences. Additionally, we could only recruit parents with a typical Dutch cultural background. Almost a quarter of the Dutch population are first- or second generation immigrants, and each could have different expectations and experiences of health care that vary with their cultural beliefs (18,35,36).

This study has several strengths regarding the credibility. First, we asked all respondents to provide feedback on a synopsis of their interview (member check 1) and asked the three respondents who constituted the representative cases to provide more detailed feedback about our interpretation of their stories (member check 2). Second, concerning investigator triangulation, two researchers with different backgrounds performed all the interviews and main analyses, while another five researchers (two GPs, a primary care researcher, epidemiologist and a medical sociologist) participated in the coding, thereby offering different theory perspectives. Due to the COVID-19 pandemic, we conducted the interviews by video call. This could have resulted in a lower credibility because in-person face-to-face interviews are considered the 'gold-standard' for qualitative research. However, recent studies indicate that both methods produce similar data volumes, topic breadths, and personal information sharing (37,38). Additionally, video interviews are more popular among respondents due to the ease of scheduling, less intimate environment, and lack of travel (38).

Using an iterative process for the data collection and analysis and continuing the data collection to saturation increased our reliability. Some readers might think a limitation of our study is the lack of data triangulation. Because we aimed to study respondent's expectations and experiences, individual in-depth interviews should have provided more valuable insights over focus group sessions or observing GP consultations.

Comparison with existing literature

In our study, parents felt reassured through different pathways, with the success of a given reassurance strategy being situation specific. Sometimes a warm GP–patient relationship, instilling feelings of safety and comfort, was sufficient to provide reassurance (affective

For peer review only - http://bmjopen.bmj.com/site/about/guidelines.xhtml

reassurance). Other times, parents needed cognitive reassurance, for example through an explanatory model for the symptoms. It is already known that specific concerns and beliefs about their symptoms (worrying cognitions) largely determine a patient's experience of cognitive reassurance (23). While some studies have argued that affective reassurance is a prerequisite to achieving cognitive reassurance (6), others state that affective reassurance only has a short-term and transient effect (8). Our findings suggest that any reassurance strategy is situation-specific, being related to both the GP–patient relationship and the disease burden.

Our findings fit with the theory of contextualising care. This states that medical decisions should be based on not only biomedical evidence but also an individualised assessment of the patient's context (39). We describe the impact of important contextual factors on the expectations and experiences of GP consultations from parental perspectives, such as emotional state (e.g., parental concern) and the relationship with their GP. Likewise, pre-existing beliefs about symptoms affected whether parents adopted the explanatory model suggested by the GP. Some parents felt like their GP did not pay enough attention to contextual factors, resulting in 'contextual errors' (Figure 2A) (39), adding to the body of evidence that contextual factors should be used in medical decision making (39,40), as recommended by Dutch GP guidelines (41,42). Research has shown that this is especially important in patients with FSS (43). Since all children in our study were diagnosed with FGID, a symptom cluster of FSS, our study can be taken to endorse the use of contextual factors in these patients.

The Dutch GP guideline advises that practitioners schedule a follow-up consultation for children with chronic abdominal pain (41). Our findings support the importance of these follow-up consultations to parents and children, not only making them feel that the GP acknowledged their disease burden but also strengthening their relationship. Hence, a planned follow-up consultation is an important step in creating an enduring doctor–patient relationship, which is a core principle of general practice that has various positive effects on both patient health and health care costs (43–46).

For peer review only - http://bmjopen.bmj.com/site/about/guidelines.xhtml

Implications for research and practice

Our findings suggest that an interrelationship exists between disease burden, the GP–patient relationship, and the reassurance given to parents and children who visit a GP for chronic GI symptoms. Our respondents identified that they have the following needs related to these themes: for the GP to acknowledge their disease burden, to search for an explanation together with the GP and to feel reassured. GPs should be aware that when they recognize and respond to these needs, they can positively influence the consultation experience of parents. Although in our study the appropriate GP response was situation specific, planning a follow-up consultation is an important first step in responding to the needs of parents and we therefore recommend GPs to do accordingly.

Further research should evaluate whether this framework also holds true for children. Consequently, future studies should investigate whether using this framework in daily practice will help GPs manage children with FGID and other FSS clusters and improve the quality of consultation experiences for parents and children.

For peer review only - http://bmjopen.bmj.com/site/about/guidelines.xhtml

Funding statement: This project was supported by ZonMW, Dutch Organization for Health Research and Development (project number 852001930).

Competing interests statement: None declared.

Authorship:

Sophie M. Ansems co-designed this study, recruited participants, performed all interviews, performed the initial analyses and interpretation of the data. She wrote the first and final draft of the manuscript.

Ilse N. Ganzevoort observed all interviews, co-performed the initial and final analysis and took part in the interpretation of the data. She revised the manuscript for important intellectual content.

Donald G. van Tol took part in the analysis and interpretation of the data. He revised the manuscript for important intellectual content.

Tryntsje Fokkema co-designed this study and took part in the analysis and interpretation of the data. She revised the manuscript for important intellectual content.

Marijke Olthof co-designed this study and took part in the interpretation of the data. She revised the manuscript for important intellectual content.

Marjolein Y. Berger co-designed this study and took part in the interpretation of the data. She revised the manuscript for important intellectual content.

Gea A. Holtman co-designed this study and took part in the interpretation of the data. She revised the manuscript for important intellectual content.

All authors approved the final version of this manuscript and agree to be accountable for all aspects of the work in ensuring that questions related to the accuracy or integrity of any part of the work are appropriately investigated and resolved.

Dr Robert Sykes (www.doctored.org.uk) provided editorial services.

For peer review only

Acknowledgements: The authors would like to thank all the respondents who volunteered and took part in this study.

Ethics approval statement: This study was approved by the Medical Research Ethics Committee of the University Medical Center Groningen (Research Register: 202000795).

Data sharing statement: Due to the confidential and sensitive nature of our data, data will not be made publicly available.

REFERENCES

1. Gieteling MJ, Lisman-van Leeuwen Y, van der Wouden JC et al. Childhood nonspecific abdominal pain in family practice: incidence, associated factors, and management. Ann Fam Med. 2011;9(4):337–43.
2. Spee LAA, Lisman-Van Leeuwen Y, Benninga MA et al. Prevalence, characteristics, and management of childhood functional abdominal pain in general practice. Scand J Prim Health Care. 2013 Dec;31(4):197–202.
3. Hyams JS, Di Lorenzo C, Saps M et al. Childhood Functional Gastrointestinal Disorders: Child/Adolescent. Gastroenterology. 2016 May 1;150(6):1456-1468.e2.
4. Bonvanie IJ, Kallesøe KH, Janssens KAM et al. Psychological Interventions for Children with Functional Somatic Symptoms: A Systematic Review and Meta-Analysis. J Pediatr. 2017 Aug 1;187:272-281.e17.
5. Olde Hartman TC, Hassink-Franke LJ, Lucassen PL et al. Explanation and relations. How do general practitioners deal with patients with persistent medically unexplained symptoms: A focus group study. BMC Fam Pract. 2009;10:68.
6. Giroldi E, Veldhuijzen W, Leijten C, Welter D et al. 'No need to worry': an exploration of general practitioners' reassuring strategies. BMC Fam Pract. 2014 Jul 7;15(1):133.
7. Terpstra T, Gol JM, Lucassen PLBJ, Houwen J et al. Explanations for medically unexplained symptoms: a qualitative study on GPs in daily practice consultations. Fam Pract. 2019 Aug 8;37(1):124–30.
8. Pincus T, Holt N, Vogel S et al. Cognitive and affective reassurance and patient outcomes in

For peer review only - http://bmjopen.bmj.com/site/about/guidelines.xhtml

primary care: A systematic review. Pain. 2013;154(11):2407–16.

9. Brodwall A, Brekke M. General practitioners' experiences with children and adolescents with functional gastro-intestinal disorders: a qualitative study in Norway. Scand J Prim Health Care. 2021;39(4):543–51.

10. Petrie KJ, Müller JT, Schirmbeck F, Donkin L et al. Effect of providing information about normal test results on patients' reassurance: Randomised controlled trial. Br Med J. 2007 Feb 17;334(7589):352–4.

11. McDonald I, Daly J, Jelinek V et al. Opening Pandora's box: the unpredictability of reassurance by a normal test result. BMJ. 1996;313(7053):329–32.

12. Ring A, Dowrick CF, Humphris GM et al. The somatising effect of clinical consultation: What patients and doctors say and do not say when patients present medically unexplained physical symptoms. In: Social Science and Medicine. Pergamon; 2005. p. 1505–15.

13. Lisman-van Leeuwen Y, Spee LAA, Benninga MA et al. Prognosis of abdominal pain in children in primary care-A prospective cohort study. Ann Fam Med. 2013;11(3):238–44.

14. Scharff L. Recurrent abdominal pain in children: a review of psychological factors and treatment. Clin Psychol Rev. 1997;17(2):145–66.

15. Ayonrinde OT, Ayonrinde OA, Adams LA et al. The relationship between abdominal pain and emotional wellbeing in children and adolescents in the Raine Study. Sci Reports 2020 101. 2020 Feb 3];10(1):1–11.

16. Brodwall A, Brekke M. Original research: Children's and adolescents' experiences with functional abdominal pain: a qualitative study in Norway. BMJ Open. 2021 Sep 13;11(9):48949.

17. Cole MAT, Qu'd D, Wild MG et al. "My Body Hates Me": A Qualitative Analysis of the Experience of Functional Nausea in Adolescent Girls and Their Mothers. Children. 2020 Aug 1;7(8).

18. Fortin S, Gauthier A, Gomez L et al. Uncertainty, culture and pathways to care in paediatric functional gastrointestinal disorders. http://dx.doi.org/101080/136484702013853026.

For peer review only - http://bmjopen.bmj.com/site/about/guidelines.xhtml

2013 Dec 1;20(3):311–23.

19. Smart S, Cottrell D. Going to the doctors: the views of mothers of children with recurrent abdominal pain. Child Care Health Dev. 2005 May 1;31(3):265–73.

20. Brodwall A, Glavin K, Lagerløv P. Parents' experience when their child has chronic abdominal pain: a qualitative study in Norway. BMJ Open. 2018;8:21066.

21. Brekke M, Brodwall A. Understanding parents' experiences of disease course and influencing factors: A 3-year follow-up qualitative study among parents of children with functional abdominal pain. BMJ Open. 2020 Aug 30;10(8).

22. van Tilburg MAL, Venepalli N, Ulshen M et al. Parents' worries about recurrent abdominal pain in children. Gastroenterol Nurs. 2006 Jan 1;29(1):50–5; quiz 56–7.

23. Giroldi E, Veldhuijzen W, Mannaerts A et al. "Doctor, please tell me it's nothing serious": an exploration of patients' worrying and reassuring cognitions using stimulated recall interviews. BMC Fam Pract. 2014 Apr 23;15(1):73.

24. Hennink M, Hutter I, Bailey A. Qualitative Research Methods. SAGE Publications Sage CA: Los Angeles, CA; 2020. Chapter 3 (page 30).

25. Tong A, Sainsbury P, Craig J. Consolidated criteria for reporting qualitative research (COREQ): a 32-item checklist for interviews and focus groups. Int J Qual Heal Care. 2007 Dec 1;19(6):349–57.

26. O'Brien BC, Harris IB, Beckman TJ et al. Standards for reporting qualitative research: a synthesis of recommendations. Acad Med. 2014;89(9):1245–51.

27. Ansems S, Berger M, van Rheenen PF et al. Effect of faecal calprotectin testing on referrals for children with chronic gastrointestinal symptoms in primary care: study protocol for a cluster randomised controlled trial. BMJ Open. 2021 Jul 23;11(7):e045444.

28. Braun V, Clarke V. Thematic Analysis. In: APA Handbook of Research Methods in Psychology: Vol 2 Resarch Designs. 2012. p. 57–71.

29. UNESCO Institute for Statistics. International Standard Classification of education - ISCED. 2011. http://uis.unesco.org/sites/default/files/documents/international-standard-

For peer review only

classification-of-education-isced-2011-en.pdf

30. Centraal Bureau voor Statistiek (CBS). Niveau ISCED 2011. https://www.cbs.nl/nl-nl/onze-diensten/methoden/classificaties/onderwijs-en-beroepen/isced/niveau-isced-2011

31. Frambach JM, van der Vleuten CPM, Durning SJ. AM last page. Quality criteria in qualitative and quantitative research. Acad Med. 2013;88(4):552.

32. Shenton AK. Strategies for ensuring trustworthiness in qualitative research projects. Educ Inf. 2004;22:63–75.

33. Thomson S, Osborn R, Squires D et al. International Profiles of Health Care Systems: Australia, Canada, Denmark, England, France, Germany, Italy, Japan, the Netherlands, New Zealand, Norway, Sweden, Switzerland, and the United States | Commonwealth Fund. 2011.

34. Fink P, Schröder A. One single diagnosis, bodily distress syndrome, succeeded to capture 10 diagnostic categories of functional somatic syndromes and somatoform disorders. J Psychosom Res. 2010 May 1;68(5):415–26.

35. Hoeveel mensen met een migratieachtergrond wonen in Nederland? https://www.cbs.nl/nl-nl/dossier/dossier-asiel-migratie-en-integratie/hoeveel-mensen-met-een-migratieachtergrond-wonen-in-nederland-

36. Nijman RG, Oostenbrink R, Dons EM et al. Parental fever attitude and management: Influence of parental ethnicity and child's age. Pediatr Emerg Care. 2010 May;26(5):339–42.

37. Krouwel M, Jolly K, Greenfield S. Comparing Skype (video calling) and in-person qualitative interview modes in a study of people with irritable bowel syndrome – an exploratory comparative analysis. BMC Med Res Methodol. 2019 Nov 29;19(1).

38. Jenner BM, Myers KC. Intimacy, rapport, and exceptional disclosure: a comparison of in-person and mediated interview contexts. Int J Soc Res Methodol. 2019 Mar 4;22(2):165–77.

For peer review only

For peer review only - http://bmjopen.bmj.com/site/about/guidelines.xhtml

39. Weiner SJ, Schwartz A. Contextual errors in medical decision making: Overlooked and understudied. Acad Med. 2016 May 1;91(5):657–62.

40. Weiner SJ, Schwartz A, Weaver F et al. Contextual errors and failures in individualizing patient care: A multicenter study. Ann Intern Med. 2010 Jul 20;153(2):69–75.

41. Gieteling MJ, van Dijk PA, de Jonge AH et al. NHG-Standaard Buikpijn bij kinderen. Huisarts Wet. 2012;55(9):404–9. ht

42. Olde Hartman TC, Blankenstein AH, Molenaar B et al. NSS. NHG Guideline on medically unexplained symptoms (MUS). Huisarts Wet. 2013;56(5):222–30.

43. Kornelsen J, Atkins C, Brownell K et al. The Meaning of Patient Experiences of Medically Unexplained Physical Symptoms: http://dx.doi.org/101177/1049732314566326. 2015 Jan 12;26(3):367–76.

44. Saltz JW, Lochner J. Interpersonal continuity of care and care outcomes: a critical review. Ann Fam Med. 2005 Mar ;3(2):159–66.

45. Haggerty JL, Reid RJ, Freeman GK et al. Continuity of care: a multidisciplinary review. BMJ. 2003 Nov 20;327(7425):1219–21.

46. Skarshaug LJ, Kaspersen SL, Bjørngaard JH et al. Original research: How does general practitioner discontinuity affect healthcare utilisation? An observational cohort study of 2.4 million Norwegians 2007–2017. BMJ Open. 2021 Feb 16;11(2):e042391.

For peer review only

Figure Legends.

Figure 1. Conceptual framework. All 3 themes (blue rectangles) influenced the expectations and experiences of parents, and their experiences were influenced by whether the GP met their expectations. GPs can influence the relationships between themes by responding to the needs of parents and children (displayed in bold, next to the arrows).

Figure 2A. Conceptual framework exemplified by Case 8. All themes are shown in red, indicating that these were negatively influenced, because the GP did not respond to the mother's needs. The 3 bold arrows indicate a strong relationship between the themes.

Figure 2B. Conceptual framework exemplified by Case 12. The themes shown in green were positively influenced because the GP responded to the father's needs. The theme shown in grey (cognitive reassurance) was not prominent during the interview. Bold arrows indicate the strong relationship between themes.

Figure 2C. Conceptual framework exemplified by Case 9. The red theme was negatively influenced because the GP did not respond to mother's needs. The grey themes were not prominent during the interview. The bold arrows indicate strong relationships between themes, and the grey arrow indicates that this relationship was not prominent during the interview.

For peer review only

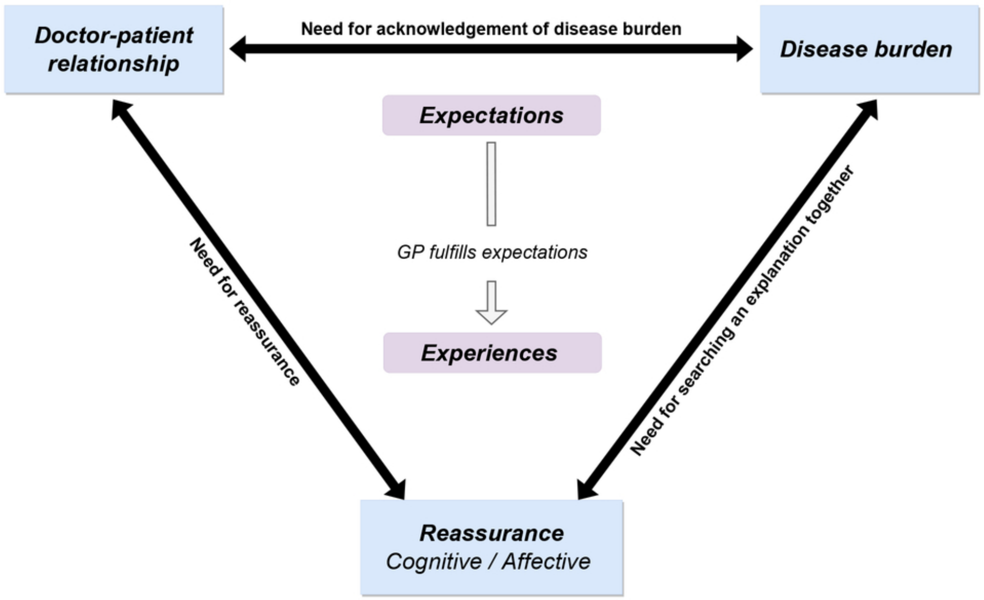

Figure 1. All 3 themes (blue rectangles) influenced the expectations and experiences of parents, and their experiences were influenced by whether the GP met their expectations. GPs can influence the relationships between themes by responding to the needs of parents and children (displayed in bold, next to the arrows).

41x24mm (600 x 600 DPI)

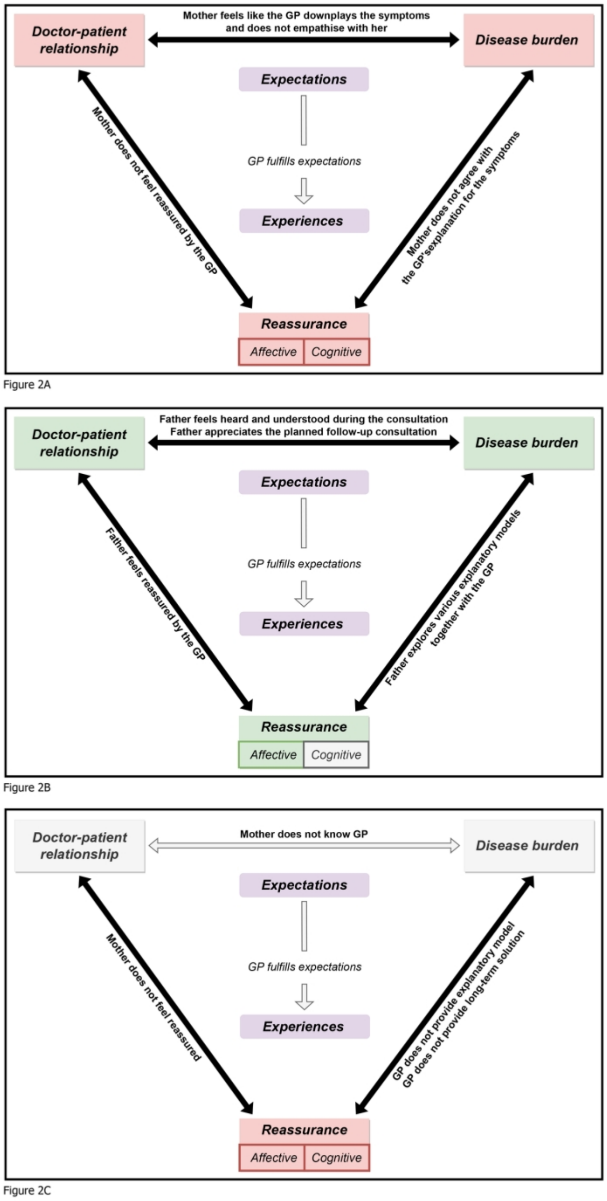

Figure 2A. Conceptual framework exemplified by Case 8. All themes are shown in red, indicating that these were negatively influenced, because the GP did not respond to the mother's needs. The 3 bold arrows indicate a strong relationship between the themes.

Figure 2B. Conceptual framework exemplified by Case 12. The themes shown in green were positively influenced because the GP responded to the father's needs. The theme shown in grey (cognitive reassurance) was not prominent during the interview. Bold arrows indicate the strong relationship between themes.

Figure 2C. Conceptual framework exemplified by Case 9. The red theme was negatively influenced because the GP did not respond to mother's needs. The grey themes were not prominent during the interview. The bold arrows indicate strong relationships between themes, and the grey arrow indicates that this relationship was not prominent during the interview.

52x103mm (600 x 600 DPI)

Supplementary File 1. Interview guide [a]

I. Introduction

- Formal introduction and explantation about the interview, including the following:
  - Study aim, "to evaluate the expectations and experiences of children with chronic gastrointestinal symptoms and their parents when visiting their GP." Explain that our goal is to improve the care provided to these children.
  - That interviews will be audio- and video-recorded for processing and analysis.
  - That all respondents will be offered a final check to indicate if they agree.
- Ask if they have any last questions or remarks before recording starts.

START RECORDING

- Explain the following:
  - That shared information will be handled confidentially and anonymously and will not be shared with the relevant GP.
  - Duration: maximum of 1 hour.
  - A synopsis of interview will be sent for a member check after the interview.

II. Opening question

Could you tell me something about your/your child's gastrointestinal symptoms?

III. Topics

1. Incentives

Why did you decide to visit your GP for the gastrointestinal symptoms?

- Expansion questions:
  - Were you concerned about the symptoms? If so, what was your biggest concern? (Serious illness / impact on daily life / school absenteeism / child's suffering.)
  - What are your views about the cause of the symptoms (serious illness / stress related / innocent)?
  - How would you describe your relationship with your GP?

2. Expectations

I would like you to go back to the day you visited the GP for the first time for the gastrointestinal symptoms. What did you hope what was going to happen?

- Possible expectations:
  - Sympathetic ear.
  - Rule out physical illness (further medical investigations).
  - Find an explanation for the symptoms.

[a] This guide has been altered slightly to make it comprehensible outside the research group

For peer review only

- Symptom relief (e.g. a change in eating pattern or medication).
- Advice on how to deal with the symptoms (whether to go to school / whether to give the child attention).

3. Experiences

How did you feel when you left the GP practice?

- Suggested feelings:
  - Taken seriously / not understood / like I waisted the GP's time / relieved / concerned/ anxious
- Expansion questions
  - What made you feel like that?
  - How did the GP contribute to this feeling?

Did the GP fulfil your expectations?

How would you score the GP's performance in the consultation on a scale from 1 to 10?

- Expansion questions: (e.g., if the answer is 7/10) what should the GP have done to turn the 7 into an 8?
- Possible improvements: Further medical investigations / referral / treatment / listen better / better explanation about the cause of the symptoms.

If you could ask the GP a question about the gastrointestinal symptoms right now, what would it be?

- Notes: Diagnosis / further steps in treatment / guidance over time

Do you now hold different views about the symptoms compared to before the GP consultation?

4. Explanation for the symptoms

What explanation did the GP provide for the symptoms? Did this explanation help you?

IV. Finishing question

Before I end the recording: is there anything else you would like to share with me?

END RECORDING

V. Important questions for all themes:

If I would ask your parents the same question, what would they answer?

If I would ask your son/daughter the same question, what would he/she answer?

[a] This guide has been altered slightly to make it comprehensible outside the research group

For peer review only - http://bmjopen.bmj.com/site/about/guidelines.xhtml

For peer review only

Supplementary File 2. Responses to the member check

Member check 1: Synopsis of the interview – All 15 respondents

Four respondents informed us that they agreed with the synopsis.

Ten respondents did not respond to the synopsis.

One mother made amendments to the synopsis clarifying her daughter's specific symptoms, why she had decided to visit the GP and how she and her husband deal with problems concerning the health of their children.

Member check 2. Conceptual framework exemplified by the interview – 3 respondents

One respondent informed us that they agreed with how their story was represented in the conceptual framework.

One respondent did not respond to our e-mail.

One respondent let us know the following via e-mail:

> *The way you have outlined the story is correct for 90%. The last part, about the relationship between me and the GP, is not completely right. When I went to the doctor for my daughter, we were seen by a representative of the GP practice. So not my own doctor. I assume her work is monitored, since my own GP called me to ask if I wanted to participate in the trial. This was in response to my visit. So, he did receive the signal. However, it is true that he also did not perform further investigations to find the cause of my daughter's symptoms. However, I do completely trust my own GP. I do not visit him often, but when I visit him, I feel taken seriously. For example, my other daughter has been admitted to the hospital due to the RS virus because he took her symptoms seriously. But this time it was a representative. Maybe it is true that my trust in her is not as high as in my own GP. But I thought that the last part in the text somewhat wronged my own GP. So that is my only remark. Besides that: fine!*

After making changes in the document clarifying that the GP discussed was not the mother's regular GP, she responded as follows:

> *The adjustment is very minimal. The reader could still interpret the story differently (as seen from my perspective). However, I do understand that this text is created with a certain aim. From that perspective, very well, use it like this. Thank you for discussing this with me.*

For peer review only

Supplementary File 2. Standards for Reporting Qualitative Research (SRQR) checklist

Standards for Reporting Qualitative Research (SRQR)*

http://www.equator-network.org/reporting-guidelines/srqr/

Title and abstract

	**Page/line no(s).**
**Title** - Concise description of the nature and topic of the study Identifying the study as qualitative or indicating the approach (e.g., ethnography, grounded theory) or data collection methods (e.g., interview, focus group) is recommended	Page 1
**Abstract** - Summary of key elements of the study using the abstract format of the intended publication; typically includes background, purpose, methods, results, and conclusions	Page 2

Introduction

**Problem formulation** - Description and significance of the problem/phenomenon studied; review of relevant theory and empirical work; problem statement	Page 5
**Purpose or research question** - Purpose of the study and specific objectives or questions	Page 5+6

Methods

**Qualitative approach and research paradigm** - Qualitative approach (e.g., ethnography, grounded theory, case study, phenomenology, narrative research) and guiding theory if appropriate; identifying the research paradigm (e.g., postpositivist, constructivist/ interpretivist) is also recommended; rationale**	Page 7 Line 3-4
**Researcher characteristics and reflexivity** - Researchers' characteristics that may influence the research, including personal attributes, qualifications/experience, relationship with participants, assumptions, and/or presuppositions; potential or actual interaction between researchers' characteristics and the research questions, approach, methods, results, and/or transferability	Page 8 Line 6-13
**Context** - Setting/site and salient contextual factors; rationale**	Page 7 Line 4-6
**Sampling strategy** - How and why research participants, documents, or events were selected; criteria for deciding when no further sampling was necessary (e.g., sampling saturation); rationale**	Page 7 Line 12-22
**Ethical issues pertaining to human subjects** - Documentation of approval by an appropriate ethics review board and participant consent, or explanation for lack thereof; other confidentiality and data security issues	Page 7 Line 22-25<br>Page 7 Line 9-10

For peer review only

**Data collection methods** - Types of data collected; details of data collection procedures including (as appropriate) start and stop dates of data collection and analysis, iterative process, triangulation of sources/methods, and modification of procedures in response to evolving study findings; rationale**	Page 7+8
**Data collection instruments and technologies** - Description of instruments (e.g., interview guides, questionnaires) and devices (e.g., audio recorders) used for data collection; if/how the instrument(s) changed over the course of the study	Page 7+8<br>Supp. File 1
**Units of study** - Number and relevant characteristics of participants, documents, or events included in the study; level of participation (could be reported in results)	Table 1<br>Page 10 3-9
**Data processing** - Methods for processing data prior to and during analysis, including transcription, data entry, data management and security, verification of data integrity, data coding, and anonymization/de-identification of excerpts	Page 7-9
**Data analysis** - Process by which inferences, themes, etc., were identified and developed, including the researchers involved in data analysis; usually references a specific paradigm or approach; rationale**	Page 8-9
**Techniques to enhance trustworthiness** - Techniques to enhance trustworthiness and credibility of data analysis (e.g., member checking, audit trail, triangulation); rationale**	Page 8 Line 19-20<br>Page 9 Line 6-8

Results/findings

**Synthesis and interpretation** - Main findings (e.g., interpretations, inferences, and themes); might include development of a theory or model, or integration with prior research or theory	Page 10-17
**Links to empirical data** - Evidence (e.g., quotes, field notes, text excerpts, photographs) to substantiate analytic findings	Page 10-17

Discussion

**Integration with prior work, implications, transferability, and contribution(s) to the field** - Short summary of main findings; explanation of how findings and conclusions connect to, support, elaborate on, or challenge conclusions of earlier scholarship; discussion of scope of application/generalizability; identification of unique contribution(s) to scholarship in a discipline or field	Page 18-21
**Limitations** - Trustworthiness and limitations of findings	Page 18-19

Other

**Conflicts of interest** - Potential sources of influence or perceived influence on study conduct and conclusions; how these were managed	Page 22
**Funding** - Sources of funding and other support; role of funders in data collection, interpretation, and reporting	Page 22

For peer review only - http://bmjopen.bmj.com/site/about/guidelines.xhtml

*The authors created the SRQR by searching the literature to identify guidelines, reporting standards, and critical appraisal criteria for qualitative research; reviewing the reference lists of retrieved sources; and contacting experts to gain feedback. The SRQR aims to improve the transparency of all aspects of qualitative research by providing clear standards for reporting qualitative research.

**The rationale should briefly discuss the justification for choosing that theory, approach, method, or technique rather than other options available, the assumptions and limitations implicit in those choices, and how those choices influence study conclusions and transferability. As appropriate, the rationale for several items might be discussed together.

Reference:

O'Brien BC, Harris IB, Beckman TJ, Reed DA, Cook DA. **Standards for reporting qualitative research: a synthesis of recommendations.** *Academic Medicine*, Vol. 89, No. 9 / Sept 2014
DOI: 10.1097/ACM.0000000000000388

For peer review only

For peer review only - http://bmjopen.bmj.com/site/about/guidelines.xhtml

SUPPLEMENTAL FILE S1: COREQ 32-ITEM CHECKLIST

Tong A, Sainsbury P, Craig J. (2007) Consolidated criteria for reporting qualitative research (COREQ): a 32-item checklist for interviews and focus groups. International Journal for Quality in Healthcare: 19:349 – 357

No. Item	Guide questions/description	Reported on Page #
Domain 1: Research team and reflexivity		
1. Inter viewer/facilitator	Which author/s conducted the interview?	8
2. Credentials	What were the researcher's credentials?	8
3. Occupation	What was their occupation at the time of the study?	8
4. Gender	Was the researcher male or female?	8
5. Experience and training	What experience or training did the researcher have?	8
6. Relationship with participants established	Was a relationship established prior to study commencement?	10
7. Participant knowledge of the interviewer	What did the participants know about the researcher?	8
8. Interviewer characteristics	What characteristics were reported about the inter viewer/facilitator?	8
Domain 2: study design		
9. Methodological orientation and Theory	What methodological orientation was stated to underpin the study?	7
10. Sampling	How were participants selected?	7
11. Method of approach	How were participants approached?	7+10
12. Sample size	How many participants were in the study?	10
13. Non-participation	How many people refused to participate or dropped out? Reasons?	10
14. Setting of data collection	Where was the data collected?	7
15. Presence of non-participants	Was anyone else present besides the participants and researchers?	8
16. Description of sample	What are the important characteristics of the sample?	Table 1
17. Interview guide	Were questions, prompts, guides provided by the authors?	Suppl. file 1
18. Repeat interviews	Were repeat interviews carried out?	NA
19. Audio/visual recording	Did the research use audio or visual recording to collect the data?	8
20. Field notes	Were field notes made during and/or	9

For peer review only

	after the inter view?	
21. Duration	What was the duration of the interviews	8
22. Data saturation	Was data saturation discussed?	7+10
23. Transcripts returned	Were transcripts returned to participants for comment and/or correction?	8+9<br>Suppl File 2
Domain 3: analysis and findings		
24. Number of data coders	How many data coders coded the data?	8+9
25. Description of the coding tree	Did authors provide a description of the coding tree?	NA
26. Derivation of themes	Were themes identified in advance or derived from the data?	7
27. Software	What software, if applicable, was used to manage the data?	9
28. Participant checking	Did participants provide feedback on the findings?	Page 10<br>Suppl File 2
29. Quotations presented	Were participant quotations presented to illustrate the themes/findings? Was each quotation identified?	13-16
30. Data and findings consistent	Was there consistency between the data presented and the findings?	10-17
31. Clarity of major themes	Were major themes clearly presented in the findings?	13-16
32. Clarity of minor themes	Is there a description of diverse cases or discussion of minor themes?	13-16

For peer review only

BMJ Open

Qualitative study evaluating the expectations and experiences of parents of children with chronic gastrointestinal symptoms visiting their general practitioner

Journal:	*BMJ Open*
Manuscript ID	bmjopen-2022-069429.R1
Article Type:	Original research
Date Submitted by the Author:	17-Apr-2023
Complete List of Authors:	Ansems, Sophie; University Medical Centre Groningen, Department of General Practice and Elderly Care Medicine<br>Ganzevoort, Ilse; University Medical Centre Groningen, General Practice and Elderly Care Medicine<br>van Tol, Donald; University Medical Centre Groningen, Department of General Practice and Elderly Care Medicine; University Medical Centre Groningen, Department of Sociology, Faculty of Behavioral and Social Sciences<br>Fokkema , Tryntsje; University Medical Centre Groningen, Department of General Practice and Elderly Care Medicine<br>Olthof, Marijke; University Medical Centre Groningen, Department of General Practice and Elderly Care Medicine<br>Berger, Marjolein; University Medical Centre Groningen, Department of General Practice and Elderly Care Medicine<br>Holtman, G.; University Medical Centre Groningen, Department of General Practice and Elderly Care Medicine
**Primary Subject Heading**:	General practice / Family practice
Secondary Subject Heading:	Gastroenterology and hepatology
Keywords:	Functional bowel disorders < GASTROENTEROLOGY, PRIMARY CARE, QUALITATIVE RESEARCH

SCHOLARONE™
Manuscripts

I, the Submitting Author has the right to grant and does grant on behalf of all authors of the Work (as defined in the below author licence), an exclusive licence and/or a non-exclusive licence for contributions from authors who are: i) UK Crown employees; ii) where BMJ has agreed a CC-BY licence shall apply, and/or iii) in accordance with the terms applicable for US Federal Government officers or employees acting as part of their official duties; on a worldwide, perpetual, irrevocable, royalty-free basis to BMJ Publishing Group Ltd ("BMJ") its licensees and where the relevant Journal is co-owned by BMJ to the co-owners of the Journal, to publish the Work in this journal and any other BMJ products and to exploit all rights, as set out in our licence.

The Submitting Author accepts and understands that any supply made under these terms is made by BMJ to the Submitting Author unless you are acting as an employee on behalf of your employer or a postgraduate student of an affiliated institution which is paying any applicable article publishing charge ("APC") for Open Access articles. Where the Submitting Author wishes to make the Work available on an Open Access basis (and intends to pay the relevant APC), the terms of reuse of such Open Access shall be governed by a Creative Commons licence – details of these licences and which Creative Commons licence will apply to this Work are set out in our licence referred to above.

Other than as permitted in any relevant BMJ Author's Self Archiving Policies, I confirm this Work has not been accepted for publication elsewhere, is not being considered for publication elsewhere and does not duplicate material already published. I confirm all authors consent to publication of this Work and authorise the granting of this licence.

For peer review only

Qualitative study evaluating the expectations and experiences of parents of children with chronic gastrointestinal symptoms visiting their general practitioner

Sophie M. Ansems [1], MD, PhD candidate | ORCID 0000000287448224 | s.m.ansems@umcg.nl

Ilse N. Ganzevoort [1], MSc, PhD candidate | i.n.ganzevoort@umcg.nl

Donald G. van Tol [1,2], PhD, Assistant Professor. | d.g.van.tol@umcg.nl

Tryntsje Fokkema [1], PhD, Researcher | tryntsjefokkema@gmail.com

Marijke Olthof [1], MD PhD, GP and researcher | marijkeolthof@gmail.com

Marjolein Y. Berger [1], MD PhD, Professor | m.y.berger@umcg.nl

Gea A. Holtman [1], PhD, Assistant Professor |ORCID 000000016579767X | g.a.holtman@umcg.nl

[1] Department of General Practice, University Medical Center Groningen, University of Groningen, the Netherlands

[2] Department of Sociology, Faculty of Behavioral and Social Sciences, University of Groningen, the Netherlands

Corresponding author: Sophie M. Ansems | Department of General Practice and Elderly Care Medicine, FA21, University of Groningen, University Medical Centre Groningen, PO Box 196, 9700 AD Groningen, the Netherlands | E-mail: s.m.ansems@umcg.nl | +31 6 14186134

Word count: 4012

Key words: functional gastrointestinal disorders, primary health care, children's health, general practitioners, qualitative research

For peer review only

ABSTRACT

Objectives: Chronic gastrointestinal symptoms are common among children and affect their daily activities and quality of life. The majority will be diagnosed with a functional gastrointestinal disorder. Effective reassurance and education are therefore key components of the physician's management. Qualitative studies have shown how parents and children experience specialist paediatric care, yet less is known about GPs, who manage most cases in the Netherlands and have a more personal and enduring relationship with their patients. Therefore, this study evaluates the expectations and experiences of parents of children visiting a GP for chronic gastrointestinal symptoms.

Design: We conducted a qualitative interview study. Online interviews were audio and video recorded, transcribed verbatim and independently analysed by the first 2 authors. Data were collected and analysed concurrently until data saturation was reached. Using thematic content analysis, we developed a conceptual framework reflecting respondent expectations and experiences. We performed a member check of the interview synopsis and the conceptual framework.

Setting: Dutch primary care.

Participants: We purposively sampled participants from a randomized controlled trial evaluating the effectiveness of faecal calprotectin testing in children with chronic gastrointestinal complaints in primary care. Thirteen parents and 2 children participated.

Results: Three key themes emerged: disease burden, GP–patient relationship, and reassurance. Often, the experienced disease burden and the pre-existing GP–patient relationship influenced expectations (e.g., for further investigations or a sympathetic ear), and when a GP fulfilled these expectations, a trusting GP–patient relationship ensued that facilitated reassurance. We found that individual needs influenced these themes and their interrelationships.

Conclusion: Insights provided by this framework could help GPs managing children with chronic gastrointestinal symptoms in daily practice and may therewith improve the consultation experience for parents. Further research should evaluate whether this framework also holds true

For peer review only - http://bmjopen.bmj.com/site/about/guidelines.xhtml

for children.

STRENGTHS AND LIMITATIONS OF THIS STUDY

- This is the first study to specifically address parental expectations of and experiences with a GP visit for paediatric chronic gastrointestinal symptoms.
- Coding and analysis was done by several researchers and two separate member checks were performed, increasing the study's credibility.
- Although our intention was to study the expectations and experiences of children as well, only 2 children participated in this study.
- Despite purposive sampling, all participants had a typical Dutch cultural background.

For peer review only

ABBREVIATIONS

COREQ	Consolidated Criteria for Reporting Qualitative Research
FGID	Functional gastrointestinal disorders
FSS	Functional somatic symptoms
GP	General Practitioner
RCT	Randomised controlled trial
SRQR	Standard Reporting of Qualitative Research

For peer review only

For peer review only - http://bmjopen.bmj.com/site/about/guidelines.xhtml

INTRODUCTION

Chronic gastrointestinal symptoms are prevalent in school-aged children, ranging from 0.3 to 19% in Western countries (1). In The Netherlands, a general practitioner (GP) will typically see 10 children with chronic gastrointestinal (GI) symptoms each year (2,3), of whom about 9 will have a functional gastrointestinal disorder (FGID) (3,4). FGID (e.g. functional constipation) is one of the symptom clusters of 'functional somatic symptoms' (FSS, i.e. symptoms that cannot be fully explained by well-defined psychiatric or somatic illness) (4,5). Other FSS clusters in children are pain, fatigue and cardiopulmonary symptoms (5). In these settings, GPs can reassure and educate parents and children through various strategies, including normalisation (6), explanation (7,8), affective reassurance (9), the exclusion of physical illness (7,10), and the provision of reassurance through normal test results (11–13). However, research has shown that 50% of children with FGID still have abdominal pain that affects daily activities 12 months after first presenting (14), while other research has shown an association between FGID and the child's quality of life, school absenteeism, anxiety, and depression (15,16).

Most current reassurance and education strategies are planned from the GP's perspective, with evidence that GPs do not always assess patient expectations and needs adequately (13). Qualitative studies among children and adolescents with FGID revealed their desire of a clear diagnosis provided by their treating physician (17–19). However, qualitative studies performed in paediatric specialist care have shown that pre-existing concerns (e.g. about serious illness or the impact of symptoms) and expectations of the doctor (e.g. to exclude physical illness or help manage pain) varied considerably among parents (20–23), creating uncertainty about the best reassurance strategy (24). Parents may also hold different views about the treating doctor, perhaps believing another professional would be better suited to treat their child or that the doctor does not consider psychologically based symptoms to be genuine (20). Other parents report that they want guidance and follow-up by their usual GP or medical professional (22). Indeed, most children with chronic GI symptoms are managed in primary care in the Netherlands (3), yet most research has taken place in paediatric specialist care (17,20–23,25,26). We

For peer review only

hypothesised that the more personal and enduring relationship between GPs and patients could influence how parents and children experience consultations. In this study, we aimed to evaluate the expectations and experiences of parents and children when visiting a GP with chronic GI symptoms through a qualitative interview study.

For peer review only

For peer review only - http://bmjopen.bmj.com/site/about/guidelines.xhtml

METHODS

Study design

We used an inductive-deductive methodology (27), meaning we used both pre-existing insights and literature as well as new insights that arose during the analysis of our data. We included parents and children living in the northern part of the Netherlands and collected data between January and June 2021. To allow personal and sensitive themes to arise, we collected data via in-depth semi-structured interviews (28). We used the Consolidated Criteria for Reporting Qualitative Research (COREQ) checklist (28) and the Standard Reporting of Qualitative Research (SRQR) checklist (28,29). This study was approved by the Medical Research Ethics Committee of the University Medical Center Groningen (Research Register: 202000795).

Respondents

Parents and children engaged in an ongoing randomised controlled trial (RCT) evaluating the clinical and cost-effectiveness of faecal calprotectin testing in primary care were eligible to take part (30). The trial included children aged 4–18 years who visited their GP with chronic abdominal pain and/or chronic diarrhoea and excluded children with a history of chronic organic GI disease or who had received endoscopic evaluation, paediatric referral for GI symptoms, or faecal calprotectin testing within the preceding 6 months. The first author approached parents and/or children by telephone after receiving informed consent for their participation in the RCT and related research.

For the present study, we used purposive sampling by age, sex (children and parents), parental educational level, and parental country of birth. Inclusion continued until data saturation, defined as being when interviews generated no new information on the research questions. All respondents received an e-mail with a patient information letter and provided digital informed consent, which consistent with Dutch law, was obtained either from parents (child age <12 years), parents and child (age 12–15 years) or the child only (age >15 years).

Data collection

An interview guide was prepared using sensitising concepts from the literature and expert

For peer review only - http://bmjopen.bmj.com/site/about/guidelines.xhtml

discussion. Sensitising concepts comprised reassurance and education strategies, parental worry cognitions, parental and child expectations, views about the GP, and experiences of the GP consultation (6,7,23,24,8,9,11–13,20–22). The interview guide contained open-ended questions concerning the incentives, expectations, and experiences related to the visit, as well as the GP's explanation for the symptoms (Supplementary File 1).

The first author, a female physician and researcher trained to perform qualitative interviews, conducted the semi-structured interviews. In the interview setting, she presented herself as a researcher and not a physician. By doing so, she prevented confusion about her role and gave the respondents the feeling they could speak freely about their GP (31,32). Another primary care researcher who is not a physician (second author, a PhD candidate studying FGID in children) observed all interviews. Before starting, we emphasised the confidential nature of the interview, that only anonymised transcripts would be kept, deleting any recordings, and that data would not be discussed with their GP. Children aged ≥12 years could decide if a parent accompanied them, but we only interviewed the parents for children aged <12 years. Interviews were held online (via Microsoft Teams) due to the COVID-19-pandemic and at a time convenient for respondents. They lasted 15–45 minutes, were audio and video recorded, and transcribed verbatim by the interviewer. Quotes were translated from Dutch to English by the first author and subsequently edited by a native English speaker and editor. Finally, the first author checked whether their meaning retained. Parents and/or children received an interview synopsis and checked whether the interpretation of the interview was correct (member check 1).

For peer review only

Analysis

Data were collected and analysed concurrently, allowing emerging themes to be incorporated and explored in subsequent interviews. Thematic ~~content~~ analysis was conducted as proposed by Braun and Clarke (33). The first and second authors coded the first three transcripts separately for standardisation. The first author then analysed the remaining transcripts and the second author independently checked the coding results before they discussed and resolved inconsistencies by consensus. Finally, two other members of the research team (a sociologist and

For peer review only - http://bmjopen.bmj.com/site/about/guidelines.xhtml

a primary care researcher) checked the coding tree. Any remaining inconsistencies and missing codes were discussed during a research team meeting.

Throughout the analysis, memos were written, including notes about the identified themes and their relationships, and provisional findings were critically discussed in research team meetings. We developed an initial conceptual framework after four meetings, following which the first author checked and confirmed whether it could be applied to each respondent's story. Further member checking was undertaken by inviting three respondents to comment on their own story. All analyses were facilitated by the use of Atlas.ti 9.1 software.

Patient and public involvement

Parents and children were involved in the interviews and its interpretation. Additionally, the results were disseminated among them, as will the manuscript once published.

For peer review only

RESULTS

Respondents

We approached 23 eligible participants by telephone, of which 18 agreed to receive further information. Two parents and one child declined to participate either for unknown reasons, personal circumstances, or not wanting to talk (1 each). Data saturation first appeared after ten interviews when we had only interviewed 1 child aged ≥12 years and 1 father. Therefore, we continued purposive recruitment of these groups for an additional five interviews to improve representation and saturation. Unfortunately, only 1 more child was recruited. All interviews took place within 2 months after the initial presentation. Table 1 shows the characteristics of the 15 respondents. The results of the initial member check for these 15 respondents are shown in Supplementary File 2 (member check 1), detailing that only one mother returned the synopsis with clarifying information.

Themes

We discovered three themes concerning the expectations and experiences of parents when visiting a GP for chronic GI symptoms: disease burden, GP–patient relationship, and reassurance, with specific needs affecting their interrelationship. Using illustrative quotations, we discuss each theme's meaning, subthemes (Table 2), and relation to the expectations and experiences of respondents, seeking to reveal the relationship between themes reinforced by respondent need (conceptual framework in Figure 1). Three cases were purposively selected for additional member check (Supplementary File 2, member check 2), as detailed in Figure 2A-2C as representative examples.

For peer review only - http://bmjopen.bmj.com/site/about/guidelines.xhtml

1 **Table 1. Parents' and children's characteristics** (*n*=15)

Characteristic	Number
Gender child, male [a]	7
Age child, years [a]	
4 -7	5
8 - 11	8
12 - 15	1
16 - 17	1
Urban area of residence [a]	5
Country of birth of both parents [b]	
The Netherlands	15
Educational level mother [b, c]	
Low	1
Intermediate	6
High	8
Educational level father [b, c]	
Low	2
Intermediate	6
High	7
Paid employment [b]	
Both parents	14
One parent	1
Marital status parents [b]	
Married / living together	11
Divorced	4
Present at interview [b]	
Child	1
Child and mother	1
Mother	8
Father	2
Both parents	3
Diagnosis after 6 months [a]	
Functional constipation	9
Irritable bowel syndrome	2
Functional constipation and functional abdominal pain	2
Functional abdominal pain	2

2 [a] Clinician reported
3 [b] Patient reported
4 [c] Educational level was divided in low (primary and lower secondary
5 education), intermediate (secondary (vocational) education) and high
6 (Bachelor's degree or higher) (34,35).
7
8
9
10

For peer review only

For peer review only - http://bmjopen.bmj.com/site/about/guidelines.xhtml

Table 2. Subthemes per theme and factors positively and negatively influencing each theme

	Subtheme	*Positive influence*	*Negative influence*
Disease burden	**Duration**	Mild symptoms	Long duration of symptoms
		Stable symptoms	Recurring symptoms
		Short symptom duration	
	**Additional symptoms Impact**		Additional symptoms (e.g. weight loss)
		No limitations due to symptoms	Symptoms impact rest of the family
		-	Child is limited in everyday life due to symptoms (e.g. school absenteeism, sports)
	**Concerns**	No concerns about symptoms	Concerns about symptoms
		No concerns because good explanation for symptoms (e.g. constipation, or runs in family)	Concerns about additional symptoms (e.g. weight loss)
		-	Concerns about impact on everyday life
	**History**	-	Parent recognise symptoms from their own medical history
		-	Other health care providers could not relieve the symptoms (e.g. psychotherapist, alternative medicine)
Doctor–patient relationship	**Pre-existing relationship**	Long-lasting doctor–patient relationship	First time at this GP
		Positive previous experience with GP	Negative previous experience with GP for chronic abdominal symptoms
	**Consultation**	Parents felt heard and understood	Not enough room in consultation for respondent’s story
		GP focused on and comforted child	Short duration of consultation
		GP showed personal interest	GP did not answer main request
		GP fulfilled expectations	GP normalised or downplayed symptoms
		GP and respondents together decided treatment plan	GP showed no empathy
	**Follow-up**	GP planned follow-up consultation	No planned follow-up consultation
		Low threshold to make new GP appointment	Parent had to contact GP about continuation
		-	GP does not take enough initiative
		-	GP provided no help besides medical care (e.g. psychosocial care)
		-	GP did not provide a solution
Cognitive reassurance	**Explanation**	Matching explanatory model	Non-matching explanatory model
		Explanatory model fulfilled the respondent’s expectation (e.g. constipation)	GP provided unclear or no explanatory model
		Respondents and GP together explored different explanatory models	GP did not seem to believe in explanatory model
		-	Respondents were unsure about explanation
	**Physical interventions**	Successful treatment (e.g. Forlax® relieved symptoms)	Unsuccessful treatment
		Physical examination confirmed diagnosis	Medical tests did not reveal an explanation
			Parents wished further medical investigation
		-	GP provided ‘quick fix’ for long-lasting problem (e.g. medication)

For peer review only - http://bmjopen.bmj.com/site/about/guidelines.xhtml

Disease burden

The disease burden experienced by parents influenced the expectations of GPs. Burden comprised the duration and severity of symptoms, the presence of additional symptoms (e.g., weight loss), the impact on everyday life, and the degree of concern about the symptoms (Table 2). Some parents experienced low disease burden (e.g., when in an early stage of symptoms), while others experienced chronic recurring GI symptoms that had limited everyday life for years.

> Mother (M8): *'For one year he was pain free and... during the last couple of months it returned. He has lost... weight, and the pain is limiting him in daily life... and in school. He arrived late to school because his belly hurt and he had to go to the toilet again. So, I just wanted further investigation.'*

Most parents with a low disease burden emphasised that they had no specific expectations of the GP.

> Mother (M6): *'Yes, exactly* [the GP fulfilled my expectations]. *Because it was not a severe situation, I did not have high expectations. I did not have a tense feeling,... so... GPs can help just by being friendly.'*

Parents had higher expectations with higher disease burdens, typically expressing concern that 'something must be wrong'. This often resulted in questions about the cause of symptoms and expectations for the GP to label symptoms, conduct further investigations, or refer to secondary care.

> Father (F1): *'At some point you are fed up... you want to know the answer, where it could come from? I know that my symptoms are clearly due to stress and everything, but I do not think that is the case for* [my daughter].*''*

> Mother (M3): *'And then I said 'You did not find anything but I would like it to be further investigated because 1) she is losing weight and 2) she just has an awful lot of pain attacks while turning pale, feeling bad, and not eating'. Her stool is almost never normal, so that cannot be right. But then, I got the response 'What investigations were you thinking of?'... I*

For peer review only - http://bmjopen.bmj.com/site/about/guidelines.xhtml

am no doctor! So... I said... 'Just send me to a paediatrician'... 'There must be a cause for this, this cannot be right'.'

Concern about symptoms varied among the parents. A couple of parents expressed concern over additional symptoms (e.g. fever), while others worried about the impact of symptoms on everyday life (e.g. school absenteeism and disturbed sleep) or the lack of explanation. By contrast, a few parents emphasised that they usually do not worry easily about their child's health and did not in this case.

Although both children mentioned that their symptoms affected them negatively during school and that they hoped the GP could find the cause and a solution, their parents had taken the initiative to consult the GP in both cases.

Doctor–patient relationship

The doctor–patient relationship comprised two elements: the pre-existing relationship (influencing both expectations and experiences) and the relationship during and after the consultation (influencing only experiences). Most parents reported a trusting relationship with their GP, with several having long-standing relationships and good previous experiences. One father expressed that his GP '*is not only a doctor, but above all, a good human being*'.

Parents emphasised the importance of feeling heard and understood by the GP during consultations. Additionally, most appreciated the GP comforting the child and directing his/her questions and explanations to the child.

Mother (M4): '*If something is wrong... he just really takes the time for you. Sometimes I am in the waiting room and the previous consultation is running late. But, actually, I find that a good sign because it means he takes the time for you. Also, for children, he really takes the time.'*

Others felt their GP downplayed the child's symptoms.

Mother (M8): *'But I do find that he could have shown a bit more empathy and... talked with my child a bit more. Because what he is feeling... is really what he is feeling. And now he has the sense that he was pretending. But it actually is... real. '*

For peer review only - http://bmjopen.bmj.com/site/about/guidelines.xhtml

Parents also felt comforted when the GP planned a follow-up consultation. This made them feel that the GP had acknowledged their concerns and the disease burden.

> Mother (M2): '*She completely examined* [my child], *we had a good conversation, and she* [said] *we will see and to call in 2 weeks to discuss it again… it was not like… manage it yourself and goodbye. Not at all.*'

Similarly, parents felt isolated when the GP did not plan a follow-up consultation.

> Mother (M1): '*If everything is ruled out* [and] *there are still symptoms, as a parent you want to do something… I would have appreciated a follow-up appointment, preferably already scheduled, so you can talk about it to find a good solution* together*. Although he cannot do anything with the test result* [everything is ruled out]*… let's keep talking…* like*... what can I help you with? Do you actually need help, or do you think you can handle it on your own?*'

When younger, the 17-year-old respondent mentioned that he found his GP intimidating. However, recent consultations for his chronic GI symptoms had changed this because the GP was considered '*friendly and neat*' towards him.

Reassurance

Parents expressed to feel reassured via two different pathways; cognitive and affective reassurance. Cognitive reassurance was achieved when the GP responded to or changed their beliefs about the symptoms (their cognitions). Whether the GP provided an explanatory model for the symptoms, and the choice of model, played prominent roles in achieving cognitive reassurance. Sometimes the GP gave an explanation that matched the parents' belief system (e.g. the GP confirmed their expectation that constipation caused the symptoms), making it relatively easy to establish cognitive reassurance. However, if their child's symptoms did not conform to the explanatory model, parents experienced insecurity about the explanation for the symptoms.

> Father (F5): '*That is the weird part because you know that the laxatives are for the constipation, but if a child suddenly has a fever and lies on the couch while having a hard time*

For peer review only - http://bmjopen.bmj.com/site/about/guidelines.xhtml

breathing… and has recovered within one hour… you do not think it is constipation… but… we did not know whether to call the GP again.'

How the GP conveyed the explanatory model also had a considerable impact on cognitive reassurance.

Child (Ch7): *'I don't know… it did feel like a bit of an oversimplification. Like… it felt like she did not know the answer herself…* [so] *she called it irritable bowel… that is how it felt.'*

This was especially the case for the two children, who both expressed that they did not understand the explanations because '*the GP talked too much*' and '*used too many difficult words*'.

Some parents emphasised that positive findings during physical examination or successful treatment made them believe in a certain explanatory model.

Mother (M4): *'Yes, I think* [the GP's final diagnosis was heartburn]*, although this was not said definitively…* [but] *since we put the bed up based on their advice,… I assume that it is related to heartburn.'*

Other parents expressed that they wanted the GP to perform more medical tests to find an explanatory model for the symptoms. However, none of the parents mentioned to feel reassured by a normal test result.

Not all parents wanted a specific explanatory model for the symptoms. Several stated that they just followed the GP's instructions (e.g. to take laxatives and return after 2 weeks with an update) and were comfortable that the GP had not provided a specific explanatory model early in the diagnostic process. In these cases, affective reassurance played a larger role, meaning parents felt reassured because the GP gave them the feeling there was no need to worry and they felt heard and understood by the GP.

Father (F14): *'She took both questions seriously… as well as our suggestions… and she found those suggestions plausible. So, she first performed a physical examination, looked at his tummy, knocked on it and she concluded that it was unlikely a purely physical cause.'*

For peer review only - http://bmjopen.bmj.com/site/about/guidelines.xhtml

Relationship between the themes and needs of parents and children

The three themes not only influenced the expectations and experiences of parents but also influenced each other (Figure 1), with this differing between interviews (see Figures 2A-2C). For example, the mother in Figure 2A suffered a high disease burden, which prompted her to seek an explanation (cognitive reassurance). In Figure 2B, the strong and long-term doctor–patient relationship made the father trust his GP when told that he should not worry about his son's symptoms (affective reassurance), without a need for an explanation for the symptoms or treatment (cognitive reassurance). By contrast, the doctor–patient relationship in Figure 2C was weak, resulting in a greater need for cognitive reassurance because the GP could not offer affective reassurance.

The relationship between the themes was also reinforced by whether the GP fulfilled or neglected the needs of parents. For example, in Figure 2B, the GP fulfilled the father's need for acknowledgement of the disease burden by planning a follow-up consultation. This, in turn, decreased the disease burden. Conversely, in Figure 2A, the GP downplayed the symptoms and disregarded the need for an explanation, increasing the disease burden and need for cognitive reassurance, respectively.

For peer review only

DISCUSSION

This qualitative interview study of the expectations and experiences when visiting a GP for chronic GI symptoms revealed three key themes: disease burden, GP–patient relationship, and reassurance. The basis of our conceptual framework is that the disease burden and pre-existing GP–patient relationship influence expectations. When a GP met parental expectations, a trusting doctor–patient relationship ensued, creating an environment that allowed affective and cognitive reassurance. Within this framework, specific needs of parents influenced the themes and their interrelationships: for the GP to acknowledge the disease burden, to search for an explanation together with the GP and to feel reassured. GPs should be aware that when they recognize and respond to these needs, they can positively influence the consultation experience of parents.

In this study, parents felt reassured through different pathways, with the success of a given reassurance strategy being situation specific. Sometimes a warm GP–patient relationship, instilling feelings of safety and comfort, was sufficient to provide reassurance (affective reassurance). Other times, parents needed cognitive reassurance, for example through an explanatory model for the symptoms. The need for an explanatory model is in concordance with a Norwegian study among parents of children with chronic abdominal pain (21). It is already known that specific concerns and beliefs about their symptoms (worrying cognitions) largely determine a patient's experience of cognitive reassurance (24). While some studies have argued that affective reassurance is a prerequisite to achieving cognitive reassurance (7), others state that affective reassurance only has a short-term and transient effect (9). Our findings suggest that any reassurance strategy is situation-specific, being related to both the GP–patient relationship and the disease burden.

Our findings fit with the theory of contextualising care, which advocates for medical decisions to be based on both biomedical evidence and the individual patient's context (36). This study found that important contextual factors, such as emotional state (e.g. parental concern) and the GP-patient relationship, affected parental expectations and experiences. Likewise, pre-existing beliefs about symptoms influenced parental adoption of the GP's explanatory model. Some

For peer review only - http://bmjopen.bmj.com/site/about/guidelines.xhtml

parents felt like their GP did not pay enough attention to contextual factors, resulting in 'contextual errors' (Figure 2A) (36), adding to the body of evidence that contextual factors should be used in medical decision making (36,37), as recommended by Dutch GP guidelines (38,39). Research has shown that this is especially important in patients with FSS (40). Since all children in our study were diagnosed with FGID, a symptom cluster of FSS, our study can be taken to endorse the use of contextual factors in these patients.

The Dutch GP guideline advises that practitioners schedule a follow-up consultation for children with chronic abdominal pain (38). Our findings support the importance of these follow-up consultations to parents and children, not only making them feel that the GP acknowledged their disease burden but also strengthening their relationship. Hence, a planned follow-up consultation is an important step in creating an enduring doctor–patient relationship, which is a core principle of general practice that has various positive effects on both patient health and health care costs (40–43).

To the best of our knowledge, there is no other study in which parents and children were interviewed about their expectations and experiences of a GP consultation when the child has chronic gastrointestinal symptoms. This is important because, in a gatekeeping system such as that in the Netherlands, the GP is the first physician to see a child with chronic GI symptoms and will typically manage most cases without referral to secondary care. Therefore, our results are transferable to other countries with gatekeeping systems, such as the United Kingdom and Canada (44). Additionally, the detailed description of our findings makes them transferable to the parents of children with other FSS (e.g. chronic fatigue) in general practice, because FSS share a similar underlying multifactorial pathophysiology (45).

This study knows some limitations. Despite the intention to interview children, only two children aged ≥12 years participated. While their expectations and experiences fit within the conceptual framework, they differed from the parent perspective. More importantly, the small number of participating children does not allow us to draw conclusions about their expectations and experiences. This implies that our findings are only transferable to parental expectations and

For peer review only - http://bmjopen.bmj.com/site/about/guidelines.xhtml

experiences. Future studies should explore children's perspectives, evaluate the framework's applicability to their expectations and experiences. Additionally, all participants had a typical Dutch cultural background. Almost a quarter of the Dutch population are first- or second generation immigrants, and each could have different expectations and experiences of health care that vary with their cultural beliefs (26,46,47). Although we purposively sampled mothers and fathers, we did not find any difference between them. The COVID-19 pandemic forced us to conduct video-interviews, which may have reduced credibility since in-person interviews are considered the 'gold-standard' for qualitative research. However, recent studies indicate that both methods produce similar data volumes, topic breadths, and personal information sharing (48,49). Additionally, video interviews are more popular among respondents due to scheduling ease, less intimate environment, and lack of travel (49).

A strength of this study is that all respondents were asked to provide feedback on a synopsis of their interview (member check 1) and the three respondents who constituted the representative cases were asked to provide more detailed feedback about our interpretation of their stories (member check 2). Another strength is that two researchers with different backgrounds performed all the interviews and main analyses, while another five researchers (two GPs, a primary care researcher, epidemiologist and a medical sociologist) participated in the coding, thereby offering different theory perspectives.

In conclusion, our findings suggest that an interrelationship exists between disease burden, the GP–patient relationship, and the reassurance given to parents and children who visit a GP for chronic GI symptoms. Although in our study the appropriate GP response was situation specific, planning a follow-up consultation is an important first step in addressing the needs of parents. We therefore recommend GPs to do accordingly, as it fosters an enduring GP–patient relationship, that is beneficial for all conditions, but particularly for patients with FSS. Further research should evaluate whether this framework also holds true for children. Additionally, it is important to explore the GP's views and experiences of these consultations. Finally, future studies should investigate whether using this framework in daily practice will help GPs manage children with

For peer review only - http://bmjopen.bmj.com/site/about/guidelines.xhtml

FGID and other FSS clusters and improve the quality of consultation experiences for parents and children.

For peer review only

For peer review only - http://bmjopen.bmj.com/site/about/guidelines.xhtml

Funding statement: This project was supported by ZonMW, Dutch Organization for Health Research and Development (project number 852001930).

Competing interests statement: None declared.

Authorship:

Sophie M. Ansems co-designed this study, recruited participants, performed all interviews, performed the initial analyses and interpretation of the data. She wrote the first and final draft of the manuscript.

Ilse N. Ganzevoort observed all interviews, co-performed the initial and final analysis and took part in the interpretation of the data. She revised the manuscript for important intellectual content.

Donald G. van Tol took part in the analysis and interpretation of the data. He revised the manuscript for important intellectual content.

Tryntsje Fokkema co-designed this study and took part in the analysis and interpretation of the data. She revised the manuscript for important intellectual content.

Marijke Olthof co-designed this study and took part in the interpretation of the data. She revised the manuscript for important intellectual content.

Marjolein Y. Berger co-designed this study and took part in the interpretation of the data. She revised the manuscript for important intellectual content.

Gea A. Holtman co-designed this study and took part in the interpretation of the data. She revised the manuscript for important intellectual content.

All authors approved the final version of this manuscript and agree to be accountable for all aspects of the work in ensuring that questions related to the accuracy or integrity of any part of the work are appropriately investigated and resolved.

Dr Robert Sykes (www.doctored.org.uk) provided editorial services.

For peer review only - http://bmjopen.bmj.com/site/about/guidelines.xhtml

Acknowledgements: The authors would like to thank all the respondents who volunteered and took part in this study.

Ethics approval statement: This study was approved by the Medical Research Ethics Committee of the University Medical Center Groningen (Research Register: 202000795).

Data sharing statement: Due to the confidential and sensitive nature of our data, data will not be made publicly available.

REFERENCES

1. Chitkara DK, Rawat DJ, Talley NJ. The Epidemiology of Childhood Recurrent Abdominal Pain in Western Countries: A Systematic Review. Am J Gastroenterol. 2005 Aug;100(8):1868–75.
2. Gieteling MJ, Lisman-van Leeuwen Y, van der Wouden JC, Schellevis FG, Berger MY. Childhood nonspecific abdominal pain in family practice: incidence, associated factors, and management. Ann Fam Med. 2011;9(4):337–43.
3. Spee LAA, Lisman-Van Leeuwen Y, Benninga MA, Bierma-Zeinstra SMA, Berger MY. Prevalence, characteristics, and management of childhood functional abdominal pain in general practice. Scand J Prim Health Care. 2013 Dec [;31(4):197–202.
4. Hyams JS, Di Lorenzo C, Saps M, Shulman RJ, Staiano A, van Tilburg M. Childhood Functional Gastrointestinal Disorders: Child/Adolescent. Gastroenterology. 2016 May 1;150(6):1456-1468.e2.
5. Bonvanie IJ, Kallesøe KH, Janssens KAM, Schröder A, Rosmalen JGM, Rask CU. Psychological Interventions for Children with Functional Somatic Symptoms: A Systematic Review and Meta-Analysis. J Pediatr. 2017 Aug 1;187:272-281.e17.
6. Olde Hartman TC, Hassink-Franke LJ, Lucassen PL, Van Spaendonck KP, Van Weel C. Explanation and relations. How do general practitioners deal with patients with persistent medically unexplained symptoms: A focus group study. BMC Fam Pract. 2009;10:68.
7. Giroldi E, Veldhuijzen W, Leijten C, Welter D, Weijden T van der, Muris J, et al. 'No need to

For peer review only - http://bmjopen.bmj.com/site/about/guidelines.xhtml

worry': an exploration of general practitioners' reassuring strategies. BMC Fam Pract. 2014 Jul 7;15(1):133.

8. Terpstra T, Gol JM, Lucassen PLBJ, Houwen J, van Dulmen S, Berger MY, et al. Explanations for medically unexplained symptoms: a qualitative study on GPs in daily practice consultations. Fam Pract. 2019 Aug 8;37(1):124–30.

9. Pincus T, Holt N, Vogel S, Underwood M, Savage R, Walsh DA, et al. Cognitive and affective reassurance and patient outcomes in primary care: A systematic review. Pain. 2013;154(11):2407–16.

10. Brodwall A, Brekke M. General practitioners' experiences with children and adolescents with functional gastro-intestinal disorders: a qualitative study in Norway. Scand J Prim Health Care. 2021;39(4):543–51.

11. Petrie KJ, Müller JT, Schirmbeck F, Donkin L, Broadbent E, Ellis CJ, et al. Effect of providing information about normal test results on patients' reassurance: Randomised controlled trial. Br Med J. 2007 Feb 17;334(7589):352–4.

12. McDonald I, Daly J, Jelinek V, Panetta F, Gutman J. Opening Pandora's box: the unpredictability of reassurance by a normal test result. BMJ. 1996;313(7053):329–32.

13. Ring A, Dowrick CF, Humphris GM, Davies J, Salmon P. The somatising effect of clinical consultation: What patients and doctors say and do not say when patients present medically unexplained physical symptoms. In: Social Science and Medicine. Pergamon; 2005. p. 1505–15.

14. Lisman-van Leeuwen Y, Spee LAA, Benninga MA, Bierma-Zeinstra SMA, Berger MY. Prognosis of abdominal pain in children in primary care-A prospective cohort study. Ann Fam Med. 2013;11(3):238–44.

15. Scharff L. Recurrent abdominal pain in children: a review of psychological factors and treatment. Clin Psychol Rev. 1997;17(2):145–66.

16. Ayonrinde OT, Ayonrinde OA, Adams LA, Sanfilippo FM, O' Sullivan TA, Robinson M, et al. The relationship between abdominal pain and emotional wellbeing in children and

For peer review only

adolescents in the Raine Study. Sci Reports 2020 101. 2020 Feb 3;10(1):1–11.

17. Brodwall A, Brekke M. Original research: Children's and adolescents' experiences with functional abdominal pain: a qualitative study in Norway. BMJ Open. 2021 Sep 13;11(9):48949.

18. Cole MAT, Qu'd D, Wild MG, Russell AC, Caillet AR, Stone AL. "My Body Hates Me": A Qualitative Analysis of the Experience of Functional Nausea in Adolescent Girls and Their Mothers. Children. 2020 Aug 1;7(8).

19. Fortin S, Gauthier A, Gomez L, Faure C, Bibeau G, Rasquin A. Uncertainty, culture and pathways to care in paediatric functional gastrointestinal disorders. http://dx.doi.org/101080/136484702013853026. 2013 Dec 1;20(3):311–23.

20. Smart S, Cottrell D. Going to the doctors: the views of mothers of children with recurrent abdominal pain. Child Care Health Dev. 2005 May 1;31(3):265–73.

21. Brodwall A, Glavin K, Lagerløv P. Parents' experience when their child has chronic abdominal pain: a qualitative study in Norway. BMJ Open. 2018;8:21066.

22. Brekke M, Brodwall A. Understanding parents' experiences of disease course and influencing factors: A 3-year follow-up qualitative study among parents of children with functional abdominal pain. BMJ Open. 2020 Aug 30;10(8).

23. van Tilburg MAL, Venepalli N, Ulshen M, Freeman KL, Levy R, Whitehead WE. Parents' worries about recurrent abdominal pain in children. Gastroenterol Nurs. 2006 Jan 1;29(1):50–5; quiz 56–7.

24. Giroldi E, Veldhuijzen W, Mannaerts A, Weijden T van der, Bareman F, Vleuten C van der. "Doctor, please tell me it's nothing serious": an exploration of patients' worrying and reassuring cognitions using stimulated recall interviews. BMC Fam Pract. 2014 Apr 23;15(1):73.

25. Cole MAT, Qu'd D, Wild MG, Russell AC, Caillet AR, Stone AL. "My Body Hates Me": A Qualitative Analysis of the Experience of Functional Nausea in Adolescent Girls and Their Mothers. Children. 2020 Aug;7(8).

For peer review only - http://bmjopen.bmj.com/site/about/guidelines.xhtml

26. Fortin S, Gauthier A, Gomez L, Faure C, Bibeau G, Rasquin A. Uncertainty, culture and pathways to care in paediatric functional gastrointestinal disorders. http://dx.doi.org/101080/136484702013853026. 2013 Dec;20(3):311–23.

27. Hennink M, Hutter I, Bailey A. Qualitative Research Methods. SAGE Publications Sage CA: Los Angeles, CA; 2020. Chapter 3: The Design Cycle.

28. Tong A, Sainsbury P, Craig J. Consolidated criteria for reporting qualitative research (COREQ): a 32-item checklist for interviews and focus groups. Int J Qual Heal Care. 2007 Dec 1;19(6):349–57.

29. O'Brien BC, Harris IB, Beckman TJ, Reed DA, Cook DA. Standards for reporting qualitative research: a synthesis of recommendations. Acad Med. 2014;89(9):1245–51.

30. Ansems S, Berger M, van Rheenen PF, Vermeulen K, Beugel G, Couwenberg M, et al. Effect of faecal calprotectin testing on referrals for children with chronic gastrointestinal symptoms in primary care: study protocol for a cluster randomised controlled trial. BMJ Open. 2021 Jul 23;11(7):e045444.

31. Collins CS, Stockton C. The Theater of Qualitative Research: The Role of the Researcher/Actor. Int J Qual Methods. 2022 May 1;21. A

32. Hennink M, Hutter I, Bailey A. Qualitative Research Methods. 2020. Chapter 7: In-depth interviews (page 127).

33. Braun V, Clarke V. Thematic Analysis. In: APA Handbook of Research Methods in Psychology: Vol 2 Resarch Designs. 2012. p. 57–71.

34. UNESCO Institute for Statistics. International Standard Classification of education - ISCED. 2011. http://uis.unesco.org/sites/default/files/documents/international-standard-classification-of-education-isced-2011-en.pdf

35. Centraal Bureau voor Statistiek (CBS). Niveau ISCED 2011. https://www.cbs.nl/nl-nl/onze-diensten/methoden/classificaties/onderwijs-en-beroepen/isced/niveau-isced-2011

36. Weiner SJ, Schwartz A. Contextual errors in medical decision making: Overlooked and

For peer review only

understudied. Acad Med. 2016 May 1;91(5):657–62.

37. Weiner SJ, Schwartz A, Weaver F, Goldberg J, Yudkowsky R, Sharma G, et al. Contextual errors and failures in individualizing patient care: A multicenter study. Ann Intern Med. 2010 Jul 20;153(2):69–75.

38. Gieteling MJ, van Dijk PA, de Jonge AH, Albeda FW, Berger MY, Burgers JS, et al. NHG-Standaard Buikpijn bij kinderen. Huisarts Wet. 2012;55(9):404–9.

39. Olde Hartman TC, Blankenstein AH, Molenaar B et al. NSS. NHG Guideline on medically unexplained symptoms (MUS). Huisarts Wet. 2013;56(5):222–30.

40. Kornelsen J, Atkins C, Brownell K, Woollard R. The Meaning of Patient Experiences of Medically Unexplained Physical Symptoms: http://dx.doi.org/101177/1049732314566326. 2015 Jan 12;26(3):367–76. A

41. Saultz JW, Lochner J. Interpersonal continuity of care and care outcomes: a critical review. Ann Fam Med. 2005 Mar;3(2):159–66.

42. Haggerty JL, Reid RJ, Freeman GK, Starfield BH, Adair CE, McKendry R. Continuity of care: a multidisciplinary review. BMJ . 2003 Nov 20;327(7425):1219–21.

43. Skarshaug LJ, Kaspersen SL, Bjørngaard JH, Pape K. Original research: How does general practitioner discontinuity affect healthcare utilisation? An observational cohort study of 2.4 million Norwegians 2007–2017. BMJ Open. 2021 Feb 16;11(2):e042391.

44. Thomson S, Osborn R, Squires D, Reed S. International Profiles of Health Care Systems: Australia, Canada, Denmark, England, France, Germany, Italy, Japan, the Netherlands, New Zealand, Norway, Sweden, Switzerland, and the United States | Commonwealth Fund . 2011.

45. Fink P, Schröder A. One single diagnosis, bodily distress syndrome, succeeded to capture 10 diagnostic categories of functional somatic syndromes and somatoform disorders. J Psychosom Res. 2010 May 1;68(5):415–26.

46. Hoeveel mensen met een migratieachtergrond wonen in Nederland? https://www.cbs.nl/nl-nl/dossier/dossier-asiel-migratie-en-integratie/hoeveel-mensen-

For peer review only - http://bmjopen.bmj.com/site/about/guidelines.xhtml

met-een-migratieachtergrond-wonen-in-nederland-

47. Nijman RG, Oostenbrink R, Dons EM, Bouwhuis CB, Moll HA. Parental fever attitude and management: Influence of parental ethnicity and child's age. Pediatr Emerg Care. 2010 May;26(5):339–42.

48. Krouwel M, Jolly K, Greenfield S. Comparing Skype (video calling) and in-person qualitative interview modes in a study of people with irritable bowel syndrome – an exploratory comparative analysis. BMC Med Res Methodol. 2019 Nov 29;19(1).

49. Jenner BM, Myers KC. Intimacy, rapport, and exceptional disclosure: a comparison of in-person and mediated interview contexts. Int J Soc Res Methodol. 2019 Mar 4;22(2):165–77.

Figure Legends.

Figure 1. Conceptual framework. All 3 themes (blue rectangles) influenced the expectations and experiences of parents, and their experiences were influenced by whether the GP met their expectations. GPs can influence the relationships between themes by responding to the needs of parents and children (displayed in bold, next to the arrows).

Figure 2A. Conceptual framework exemplified by Case 8. All themes are shown in red, indicating that these were negatively influenced, because the GP did not respond to the mother's needs. The 3 bold arrows indicate a strong relationship between the themes.

Figure 2B. Conceptual framework exemplified by Case 12. The themes shown in green were positively influenced because the GP responded to the father's needs. The theme shown in grey (cognitive reassurance) was not prominent during the interview. Bold arrows indicate the strong relationship between themes.

Figure 2C. Conceptual framework exemplified by Case 9. The red theme was negatively influenced because the GP did not respond to mother's needs. The grey themes were not prominent during the interview. The bold arrows indicate strong relationships between themes, and the grey arrow indicates that this relationship was not prominent during the interview.

For peer review only - http://bmjopen.bmj.com/site/about/guidelines.xhtml

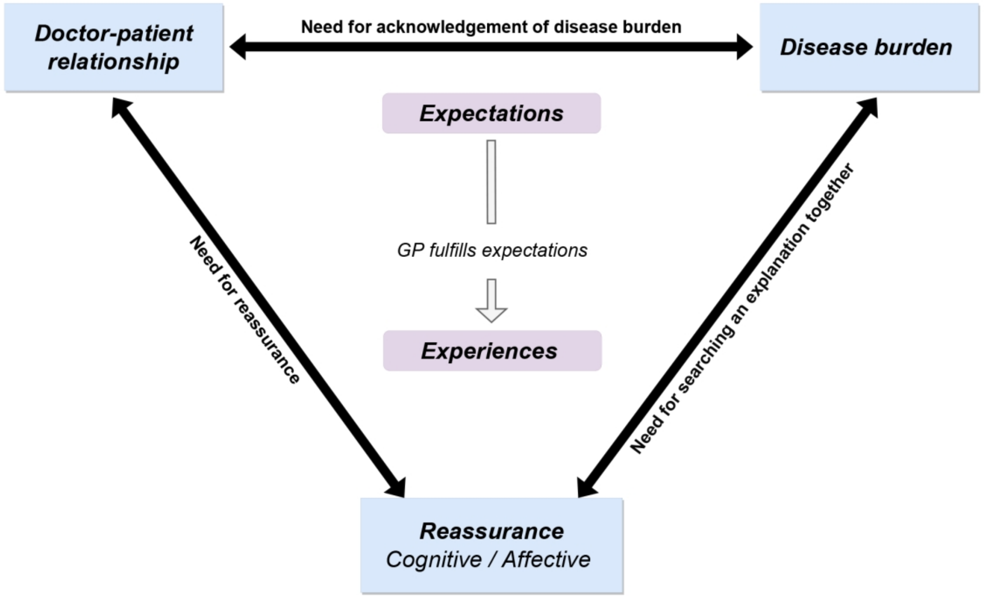

Figure 1. All 3 themes (blue rectangles) influenced the expectations and experiences of parents, and their experiences were influenced by whether the GP met their expectations. GPs can influence the relationships between themes by responding to the needs of parents and children (displayed in bold, next to the arrows).

41x24mm (1200 x 1200 DPI)

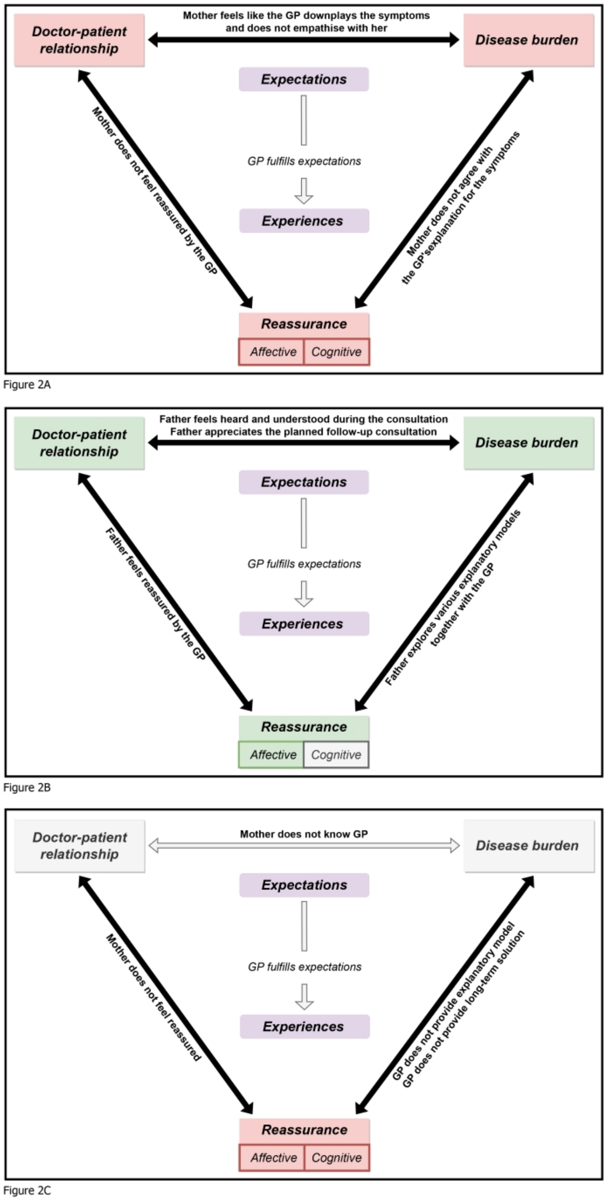

Figure 2A. Conceptual framework exemplified by Case 8. All themes are shown in red, indicating that these were negatively influenced, because the GP did not respond to the mother's needs. The 3 bold arrows indicate a strong relationship between the themes.
Figure 2B. Conceptual framework exemplified by Case 12. The themes shown in green were positively influenced because the GP responded to the father's needs. The theme shown in grey (cognitive reassurance) was not prominent during the interview. Bold arrows indicate the strong relationship between themes.
Figure 2C. Conceptual framework exemplified by Case 9. The red theme was negatively influenced because the GP did not respond to mother's needs. The grey themes were not prominent during the interview. The bold arrows indicate strong relationships between themes, and the grey arrow indicates that this relationship was not prominent during the interview.

52x103mm (1200 x 1200 DPI)

For peer review only - http://bmjopen.bmj.com/site/about/guidelines.xhtml

Supplementary File 1. Interview guide [a]

I. Introduction

- Formal introduction and explantation about the interview, including the following:
  - Study aim, "to evaluate the expectations and experiences of children with chronic gastrointestinal symptoms and their parents when visiting their GP." Explain that our goal is to improve the care provided to these children.
  - That interviews will be audio- and video-recorded for processing and analysis.
  - That all respondents will be offered a final check to indicate if they agree.
- Ask if they have any last questions or remarks before recording starts.

START RECORDING

- Explain the following:
  - That shared information will be handled confidentially and anonymously and will not be shared with the relevant GP.
  - Duration: maximum of 1 hour.
  - A synopsis of interview will be sent for a member check after the interview.

II. Opening question

Could you tell me something about your/your child's gastrointestinal symptoms?

III. Topics

1. Incentives

Why did you decide to visit your GP for the gastrointestinal symptoms?

- Expansion questions:
  - Were you concerned about the symptoms? If so, what was your biggest concern? (Serious illness / impact on daily life / school absenteeism / child's suffering.)
  - What are your views about the cause of the symptoms (serious illness / stress related / innocent)?
  - How would you describe your relationship with your GP?

2. Expectations

I would like you to go back to the day you visited the GP for the first time for the gastrointestinal symptoms. What did you hope what was going to happen?

- Possible expectations:
  - Sympathetic ear.
  - Rule out physical illness (further medical investigations).
  - Find an explanation for the symptoms.

[a] This guide has been altered slightly to make it comprehensible outside the research group

- Symptom relief (e.g. a change in eating pattern or medication).
- Advice on how to deal with the symptoms (whether to go to school / whether to give the child attention).

3. Experiences

How did you feel when you left the GP practice?

- Suggested feelings:
  - Taken seriously / not understood / like I waisted the GP's time / relieved / concerned/ anxious
- Expansion questions
  - What made you feel like that?
  - How did the GP contribute to this feeling?

Did the GP fulfil your expectations?

How would you score the GP's performance in the consultation on a scale from 1 to 10?

- Expansion questions: (e.g., if the answer is 7/10) what should the GP have done to turn the 7 into an 8?
- Possible improvements: Further medical investigations / referral / treatment / listen better / better explanation about the cause of the symptoms.

If you could ask the GP a question about the gastrointestinal symptoms right now, what would it be?

- Notes: Diagnosis / further steps in treatment / guidance over time

Do you now hold different views about the symptoms compared to before the GP consultation?

4. Explanation for the symptoms

What explanation did the GP provide for the symptoms? Did this explanation help you?

IV. Finishing question

Before I end the recording: is there anything else you would like to share with me?

END RECORDING

V. Important questions for all themes:

If I would ask your parents the same question, what would they answer?

If I would ask your son/daughter the same question, what would he/she answer?

[a] This guide has been altered slightly to make it comprehensible outside the research group

For peer review only

For peer review only

Supplementary File 2. Responses to the member check

Member check 1: Synopsis of the interview – All 15 respondents

Four respondents informed us that they agreed with the synopsis.

Ten respondents did not respond to the synopsis.

One mother made amendments to the synopsis clarifying her daughter's specific symptoms, why she had decided to visit the GP and how she and her husband deal with problems concerning the health of their children.

Member check 2. Conceptual framework exemplified by the interview – 3 respondents

One respondent informed us that they agreed with how their story was represented in the conceptual framework.

One respondent did not respond to our e-mail.

One respondent let us know the following via e-mail:

> *The way you have outlined the story is correct for 90%. The last part, about the relationship between me and the GP, is not completely right. When I went to the doctor for my daughter, we were seen by a representative of the GP practice. So not my own doctor. I assume her work is monitored, since my own GP called me to ask if I wanted to participate in the trial. This was in response to my visit. So, he did receive the signal. However, it is true that he also did not perform further investigations to find the cause of my daughter's symptoms. However, I do completely trust my own GP. I do not visit him often, but when I visit him, I feel taken seriously. For example, my other daughter has been admitted to the hospital due to the RS virus because he took her symptoms seriously. But this time it was a representative. Maybe it is true that my trust in her is not as high as in my own GP. But I thought that the last part in the text somewhat wronged my own GP. So that is my only remark. Besides that: fine!*

After making changes in the document clarifying that the GP discussed was not the mother's regular GP, she responded as follows:

> *The adjustment is very minimal. The reader could still interpret the story differently (as seen from my perspective). However, I do understand that this text is created with a certain aim. From that perspective, very well, use it like this. Thank you for discussing this with me.*

For peer review only

Supplementary File 2. Standards for Reporting Qualitative Research (SRQR) checklist

Standards for Reporting Qualitative Research (SRQR)*

http://www.equator-network.org/reporting-guidelines/srqr/

Page/line no(s).

Title and abstract

**Title** - Concise description of the nature and topic of the study Identifying the study as qualitative or indicating the approach (e.g., ethnography, grounded theory) or data collection methods (e.g., interview, focus group) is recommended	Page 1
**Abstract** - Summary of key elements of the study using the abstract format of the intended publication; typically includes background, purpose, methods, results, and conclusions	Page 2

Introduction

**Problem formulation** - Description and significance of the problem/phenomenon studied; review of relevant theory and empirical work; problem statement	Page 5
**Purpose or research question** - Purpose of the study and specific objectives or questions	Page 5+6

Methods

**Qualitative approach and research paradigm** - Qualitative approach (e.g., ethnography, grounded theory, case study, phenomenology, narrative research) and guiding theory if appropriate; identifying the research paradigm (e.g., postpositivist, constructivist/ interpretivist) is also recommended; rationale**	Page 7 Line 3-4
**Researcher characteristics and reflexivity** - Researchers' characteristics that may influence the research, including personal attributes, qualifications/experience, relationship with participants, assumptions, and/or presuppositions; potential or actual interaction between researchers' characteristics and the research questions, approach, methods, results, and/or transferability	Page 8 Line 6-13
**Context** - Setting/site and salient contextual factors; rationale**	Page 7 Line 4-6
**Sampling strategy** - How and why research participants, documents, or events were selected; criteria for deciding when no further sampling was necessary (e.g., sampling saturation); rationale**	Page 7 Line 12-22
**Ethical issues pertaining to human subjects** - Documentation of approval by an appropriate ethics review board and participant consent, or explanation for lack thereof; other confidentiality and data security issues	Page 7 Line 22-25<br>Page 7 Line 9-10

For peer review only - http://bmjopen.bmj.com/site/about/guidelines.xhtml

**Data collection methods** - Types of data collected; details of data collection procedures including (as appropriate) start and stop dates of data collection and analysis, iterative process, triangulation of sources/methods, and modification of procedures in response to evolving study findings; rationale**	Page 7+8
**Data collection instruments and technologies** - Description of instruments (e.g., interview guides, questionnaires) and devices (e.g., audio recorders) used for data collection; if/how the instrument(s) changed over the course of the study	Page 7+8<br>Supp. File 1
**Units of study** - Number and relevant characteristics of participants, documents, or events included in the study; level of participation (could be reported in results)	Table 1<br>Page 10 3-9
**Data processing** - Methods for processing data prior to and during analysis, including transcription, data entry, data management and security, verification of data integrity, data coding, and anonymization/de-identification of excerpts	Page 7-9
**Data analysis** - Process by which inferences, themes, etc., were identified and developed, including the researchers involved in data analysis; usually references a specific paradigm or approach; rationale**	Page 8-9
**Techniques to enhance trustworthiness** - Techniques to enhance trustworthiness and credibility of data analysis (e.g., member checking, audit trail, triangulation); rationale**	Page 8 Line 19-20<br>Page 9 Line 6-8

Results/findings

**Synthesis and interpretation** - Main findings (e.g., interpretations, inferences, and themes); might include development of a theory or model, or integration with prior research or theory	Page 10-17
**Links to empirical data** - Evidence (e.g., quotes, field notes, text excerpts, photographs) to substantiate analytic findings	Page 10-17

Discussion

**Integration with prior work, implications, transferability, and contribution(s) to the field** - Short summary of main findings; explanation of how findings and conclusions connect to, support, elaborate on, or challenge conclusions of earlier scholarship; discussion of scope of application/generalizability; identification of unique contribution(s) to scholarship in a discipline or field	Page 18-21
**Limitations** - Trustworthiness and limitations of findings	Page 18-19

Other

**Conflicts of interest** - Potential sources of influence or perceived influence on study conduct and conclusions; how these were managed	Page 22
**Funding** - Sources of funding and other support; role of funders in data collection, interpretation, and reporting	Page 22

For peer review only - http://bmjopen.bmj.com/site/about/guidelines.xhtml

*The authors created the SRQR by searching the literature to identify guidelines, reporting standards, and critical appraisal criteria for qualitative research; reviewing the reference lists of retrieved sources; and contacting experts to gain feedback. The SRQR aims to improve the transparency of all aspects of qualitative research by providing clear standards for reporting qualitative research.

**The rationale should briefly discuss the justification for choosing that theory, approach, method, or technique rather than other options available, the assumptions and limitations implicit in those choices, and how those choices influence study conclusions and transferability. As appropriate, the rationale for several items might be discussed together.

Reference:

O'Brien BC, Harris IB, Beckman TJ, Reed DA, Cook DA. **Standards for reporting qualitative research: a synthesis of recommendations.** *Academic Medicine*, Vol. 89, No. 9 / Sept 2014
DOI: 10.1097/ACM.0000000000000388

For peer review only

For peer review only - http://bmjopen.bmj.com/site/about/guidelines.xhtml

SUPPLEMENTAL FILE S1: COREQ 32-ITEM CHECKLIST

Tong A, Sainsbury P, Craig J. (2007) Consolidated criteria for reporting qualitative research (COREQ): a 32-item checklist for interviews and focus groups. International Journal for Quality in Healthcare: 19:349 – 357

No. Item	Guide questions/description	Reported on Page #
Domain 1: Research team and reflexivity		
1. Inter viewer/facilitator	Which author/s conducted the interview?	8
2. Credentials	What were the researcher's credentials?	8
3. Occupation	What was their occupation at the time of the study?	8
4. Gender	Was the researcher male or female?	8
5. Experience and training	What experience or training did the researcher have?	8
6. Relationship with participants established	Was a relationship established prior to study commencement?	10
7. Participant knowledge of the interviewer	What did the participants know about the researcher?	8
8. Interviewer characteristics	What characteristics were reported about the inter viewer/facilitator?	8
Domain 2: study design		
9. Methodological orientation and Theory	What methodological orientation was stated to underpin the study?	7
10. Sampling	How were participants selected?	7
11. Method of approach	How were participants approached?	7+10
12. Sample size	How many participants were in the study?	10
13. Non-participation	How many people refused to participate or dropped out? Reasons?	10
14. Setting of data collection	Where was the data collected?	7
15. Presence of non-participants	Was anyone else present besides the participants and researchers?	8
16. Description of sample	What are the important characteristics of the sample?	Table 1
17. Interview guide	Were questions, prompts, guides provided by the authors?	Suppl. file 1
18. Repeat interviews	Were repeat interviews carried out?	NA
19. Audio/visual recording	Did the research use audio or visual recording to collect the data?	8
20. Field notes	Were field notes made during and/or	9

For peer review only

	after the inter view?	
21. Duration	What was the duration of the interviews	8
22. Data saturation	Was data saturation discussed?	7+10
23. Transcripts returned	Were transcripts returned to participants for comment and/or correction?	8+9<br>Suppl File 2
Domain 3: analysis and findings		
24. Number of data coders	How many data coders coded the data?	8+9
25. Description of the coding tree	Did authors provide a description of the coding tree?	NA
26. Derivation of themes	Were themes identified in advance or derived from the data?	7
27. Software	What software, if applicable, was used to manage the data?	9
28. Participant checking	Did participants provide feedback on the findings?	Page 10<br>Suppl File 2
29. Quotations presented	Were participant quotations presented to illustrate the themes/findings? Was each quotation identified?	13-16
30. Data and findings consistent	Was there consistency between the data presented and the findings?	10-17
31. Clarity of major themes	Were major themes clearly presented in the findings?	13-16
32. Clarity of minor themes	Is there a description of diverse cases or discussion of minor themes?	13-16

For peer review only

BMJ Open

Qualitative study evaluating the expectations and experiences of Dutch parents of children with chronic gastrointestinal symptoms visiting their general practitioner

Journal:	*BMJ Open*
Manuscript ID	bmjopen-2022-069429.R2
Article Type:	Original research
Date Submitted by the Author:	01-May-2023
Complete List of Authors:	Ansems, Sophie; University Medical Centre Groningen, Department of General Practice and Elderly Care Medicine<br>Ganzevoort, Ilse; University Medical Centre Groningen, General Practice and Elderly Care Medicine<br>van Tol, Donald; University Medical Centre Groningen, Department of General Practice and Elderly Care Medicine; University Medical Centre Groningen, Department of Sociology, Faculty of Behavioral and Social Sciences<br>Fokkema , Tryntsje; University Medical Centre Groningen, Department of General Practice and Elderly Care Medicine<br>Olthof, Marijke; University Medical Centre Groningen, Department of General Practice and Elderly Care Medicine<br>Berger, Marjolein; University Medical Centre Groningen, Department of General Practice and Elderly Care Medicine<br>Holtman, G.; University Medical Centre Groningen, Department of General Practice and Elderly Care Medicine
**Primary Subject Heading**:	General practice / Family practice
Secondary Subject Heading:	Gastroenterology and hepatology
Keywords:	Functional bowel disorders < GASTROENTEROLOGY, PRIMARY CARE, QUALITATIVE RESEARCH

SCHOLARONE™
Manuscripts

I, the Submitting Author has the right to grant and does grant on behalf of all authors of the Work (as defined in the below author licence), an exclusive licence and/or a non-exclusive licence for contributions from authors who are: i) UK Crown employees; ii) where BMJ has agreed a CC-BY licence shall apply, and/or iii) in accordance with the terms applicable for US Federal Government officers or employees acting as part of their official duties; on a worldwide, perpetual, irrevocable, royalty-free basis to BMJ Publishing Group Ltd ("BMJ") its licensees and where the relevant Journal is co-owned by BMJ to the co-owners of the Journal, to publish the Work in this journal and any other BMJ products and to exploit all rights, as set out in our licence.

The Submitting Author accepts and understands that any supply made under these terms is made by BMJ to the Submitting Author unless you are acting as an employee on behalf of your employer or a postgraduate student of an affiliated institution which is paying any applicable article publishing charge ("APC") for Open Access articles. Where the Submitting Author wishes to make the Work available on an Open Access basis (and intends to pay the relevant APC), the terms of reuse of such Open Access shall be governed by a Creative Commons licence – details of these licences and which Creative Commons licence will apply to this Work are set out in our licence referred to above.

Other than as permitted in any relevant BMJ Author's Self Archiving Policies, I confirm this Work has not been accepted for publication elsewhere, is not being considered for publication elsewhere and does not duplicate material already published. I confirm all authors consent to publication of this Work and authorise the granting of this licence.

For peer review only

Qualitative study evaluating the expectations and experiences of Dutch parents of children with chronic gastrointestinal symptoms visiting their general practitioner

Sophie M. Ansems [1], MD, PhD candidate | ORCID 0000000287448224 | s.m.ansems@umcg.nl

Ilse N. Ganzevoort [1], MSc, PhD candidate | i.n.ganzevoort@umcg.nl

Donald G. van Tol [1,2], PhD, Assistant Professor. | d.g.van.tol@umcg.nl

Tryntsje Fokkema [1], PhD, Researcher | tryntsjefokkema@gmail.com

Marijke Olthof [1], MD PhD, GP and researcher | marijkeolthof@gmail.com

Marjolein Y. Berger [1], MD PhD, Professor | m.y.berger@umcg.nl

Gea A. Holtman [1], PhD, Assistant Professor |ORCID 000000016579767X | g.a.holtman@umcg.nl

[1] Department of General Practice, University Medical Center Groningen, University of Groningen, the Netherlands

[2] Department of Sociology, Faculty of Behavioral and Social Sciences, University of Groningen, the Netherlands

Corresponding author: Sophie M. Ansems | Department of General Practice and Elderly Care Medicine, FA21, University of Groningen, University Medical Centre Groningen, PO Box 196, 9700 AD Groningen, the Netherlands | E-mail: s.m.ansems@umcg.nl | +31 6 14186134

Word count: 4012

Key words: functional gastrointestinal disorders, primary health care, children's health, general practitioners, qualitative research

For peer review only

ABSTRACT

Objectives: Chronic gastrointestinal symptoms are common among children and affect their daily activities and quality of life. The majority will be diagnosed with a functional gastrointestinal disorder. Effective reassurance and education are therefore key components of the physician's management. Qualitative studies have shown how parents and children experience specialist paediatric care, yet less is known about GPs, who manage most cases in the Netherlands and have a more personal and enduring relationship with their patients. Therefore, this study evaluates the expectations and experiences of parents of children visiting a GP for chronic gastrointestinal symptoms.

Design: We conducted a qualitative interview study. Online interviews were audio and video recorded, transcribed verbatim and independently analysed by the first 2 authors. Data were collected and analysed concurrently until data saturation was reached. Using thematic content analysis, we developed a conceptual framework reflecting respondent expectations and experiences. We performed a member check of the interview synopsis and the conceptual framework.

Setting: Dutch primary care.

Participants: We purposively sampled participants from a randomized controlled trial evaluating the effectiveness of faecal calprotectin testing in children with chronic gastrointestinal complaints in primary care. Thirteen parents and 2 children participated.

Results: Three key themes emerged: disease burden, GP–patient relationship, and reassurance. Often, the experienced disease burden and the pre-existing GP–patient relationship influenced expectations (e.g., for further investigations or a sympathetic ear), and when a GP fulfilled these expectations, a trusting GP–patient relationship ensued that facilitated reassurance. We found that individual needs influenced these themes and their interrelationships.

Conclusion: Insights provided by this framework could help GPs managing children with chronic gastrointestinal symptoms in daily practice and may therewith improve the consultation experience for parents. Further research should evaluate whether this framework also holds true

For peer review only - http://bmjopen.bmj.com/site/about/guidelines.xhtml

for children.

STRENGTHS AND LIMITATIONS OF THIS STUDY

- This is the first study to specifically address parental expectations of and experiences with a GP visit for paediatric chronic gastrointestinal symptoms.
- Coding and analysis was done by several researchers and two separate member checks were performed, increasing the study's credibility.
- Although our intention was to study the expectations and experiences of children as well, only 2 children participated in this study.
- Despite purposive sampling, all participants had a typical Dutch cultural background.

For peer review only

ABBREVIATIONS

COREQ	Consolidated Criteria for Reporting Qualitative Research
FGID	Functional gastrointestinal disorders
FSS	Functional somatic symptoms
GP	General Practitioner
RCT	Randomised controlled trial
SRQR	Standard Reporting of Qualitative Research

For peer review only

For peer review only - http://bmjopen.bmj.com/site/about/guidelines.xhtml

INTRODUCTION

Chronic gastrointestinal symptoms are prevalent in school-aged children, ranging from 0.3 to 19% in Western countries (1). In The Netherlands, a general practitioner (GP) will typically see 10 children with chronic gastrointestinal (GI) symptoms each year (2,3), of whom about 9 will have a functional gastrointestinal disorder (FGID) (3,4). FGID (e.g. functional constipation) is one of the symptom clusters of 'functional somatic symptoms' (FSS, i.e. symptoms that cannot be fully explained by well-defined psychiatric or somatic illness) (4,5). Other FSS clusters in children are pain, fatigue and cardiopulmonary symptoms (5). In these settings, GPs can reassure and educate parents and children through various strategies, including normalisation (6), explanation (7,8), affective reassurance (9), the exclusion of physical illness (7,10), and the provision of reassurance through normal test results (11–13). However, research has shown that 50% of children with FGID still have abdominal pain that affects daily activities 12 months after first presenting (14), while other research has shown an association between FGID and the child's quality of life, school absenteeism, anxiety, and depression (15,16).

Most current reassurance and education strategies are planned from the GP's perspective, with evidence that GPs do not always assess patient expectations and needs adequately (13). Qualitative studies among children and adolescents with FGID revealed their desire of a clear diagnosis provided by their treating physician (17–19). However, qualitative studies performed in paediatric specialist care have shown that pre-existing concerns (e.g. about serious illness or the impact of symptoms) and expectations of the doctor (e.g. to exclude physical illness or help manage pain) varied considerably among parents (20–23), creating uncertainty about the best reassurance strategy (24). Parents may also hold different views about the treating doctor, perhaps believing another professional would be better suited to treat their child or that the doctor does not consider psychologically based symptoms to be genuine (20). Other parents report that they want guidance and follow-up by their usual GP or medical professional (22). Indeed, most children with chronic GI symptoms are managed in primary care in the Netherlands (3), yet most research has taken place in paediatric specialist care (17,20–23,25,26). We

For peer review only

hypothesised that the more personal and enduring relationship between GPs and patients could influence how parents and children experience consultations. In this study, we aimed to evaluate the expectations and experiences of parents and children when visiting a GP with chronic GI symptoms through a qualitative interview study.

For peer review only

For peer review only - http://bmjopen.bmj.com/site/about/guidelines.xhtml

METHODS

Study design

We used an inductive-deductive methodology (27), meaning we used both pre-existing insights and literature as well as new insights that arose during the analysis of our data. We included parents and children living in the northern part of the Netherlands and collected data between January and June 2021. To allow personal and sensitive themes to arise, we collected data via in-depth semi-structured interviews (28). We used the Consolidated Criteria for Reporting Qualitative Research (COREQ) checklist (28) and the Standard Reporting of Qualitative Research (SRQR) checklist (28,29). This study was approved by the Medical Research Ethics Committee of the University Medical Center Groningen (Research Register: 202000795).

Respondents

Parents and children engaged in an ongoing randomised controlled trial (RCT) evaluating the clinical and cost-effectiveness of faecal calprotectin testing in primary care were eligible to take part (30) (The Netherlands Trial Register: NL7690). The trial included children aged 4–18 years who visited their GP with chronic abdominal pain and/or chronic diarrhoea and excluded children with a history of chronic organic GI disease or who had received endoscopic evaluation, paediatric referral for GI symptoms, or faecal calprotectin testing within the preceding 6 months. The first author approached parents and/or children by telephone after receiving informed consent for their participation in the RCT and related research.

For the present study, we used purposive sampling by age, sex (children and parents), parental educational level, and parental country of birth. Inclusion continued until data saturation, defined as being when interviews generated no new information on the research questions. All respondents received an e-mail with a patient information letter and provided digital informed consent, which consistent with Dutch law, was obtained either from parents (child age <12 years), parents and child (age 12–15 years) or the child only (age >15 years).

Data collection

An interview guide was prepared using sensitising concepts from the literature and expert

For peer review only - http://bmjopen.bmj.com/site/about/guidelines.xhtml

discussion. Sensitising concepts comprised reassurance and education strategies, parental worry cognitions, parental and child expectations, views about the GP, and experiences of the GP consultation (6,7,23,24,8,9,11–13,20–22). The interview guide contained open-ended questions concerning the incentives, expectations, and experiences related to the visit, as well as the GP's explanation for the symptoms (Supplementary File 1).

The first author, a female physician and researcher trained to perform qualitative interviews, conducted the semi-structured interviews. In the interview setting, she presented herself as a researcher and not a physician. By doing so, she prevented confusion about her role and gave the respondents the feeling they could speak freely about their GP (31,32). Another primary care researcher who is not a physician (second author, a PhD candidate studying FGID in children) observed all interviews. Before starting, we emphasised the confidential nature of the interview, that only anonymised transcripts would be kept, deleting any recordings, and that data would not be discussed with their GP. Children aged ≥12 years could decide if a parent accompanied them, but we only interviewed the parents for children aged <12 years. Interviews were held online (via Microsoft Teams) due to the COVID-19-pandemic and at a time convenient for respondents. They lasted 15–45 minutes, were audio and video recorded, and transcribed verbatim by the interviewer. Quotes were translated from Dutch to English by the first author and subsequently edited by a native English speaker and editor. Finally, the first author checked whether their meaning retained. Parents and/or children received an interview synopsis and checked whether the interpretation of the interview was correct (member check 1).

For peer review only

Analysis

Data were collected and analysed concurrently, allowing emerging themes to be incorporated and explored in subsequent interviews. Thematic ~~content~~ analysis was conducted as proposed by Braun and Clarke (33). The first and second authors coded the first three transcripts separately for standardisation. The first author then analysed the remaining transcripts and the second author independently checked the coding results before they discussed and resolved inconsistencies by consensus. Finally, two other members of the research team (a sociologist and

For peer review only - http://bmjopen.bmj.com/site/about/guidelines.xhtml

a primary care researcher) checked the coding tree. Any remaining inconsistencies and missing codes were discussed during a research team meeting.

Throughout the analysis, memos were written, including notes about the identified themes and their relationships, and provisional findings were critically discussed in research team meetings. We developed an initial conceptual framework after four meetings, following which the first author checked and confirmed whether it could be applied to each respondent's story. Further member checking was undertaken by inviting three respondents to comment on their own story. All analyses were facilitated by the use of Atlas.ti 9.1 software.

Patient and public involvement

Parents and children were involved in the interviews and its interpretation. Additionally, the results were disseminated among them, as will the manuscript once published.

For peer review only

RESULTS

Respondents

We approached 23 eligible participants by telephone, of which 18 agreed to receive further information. Two parents and one child declined to participate either for unknown reasons, personal circumstances, or not wanting to talk (1 each). Data saturation first appeared after ten interviews when we had only interviewed 1 child aged ≥12 years and 1 father. Therefore, we continued purposive recruitment of these groups for an additional five interviews to improve representation and saturation. Unfortunately, only 1 more child was recruited. All interviews took place within 2 months after the initial presentation. Table 1 shows the characteristics of the 15 respondents. The results of the initial member check for these 15 respondents are shown in Supplementary File 2 (member check 1), detailing that only one mother returned the synopsis with clarifying information.

Themes

We discovered three themes concerning the expectations and experiences of parents when visiting a GP for chronic GI symptoms: disease burden, GP–patient relationship, and reassurance, with specific needs affecting their interrelationship. Using illustrative quotations, we discuss each theme's meaning, subthemes (Table 2), and relation to the expectations and experiences of respondents, seeking to reveal the relationship between themes reinforced by respondent need (conceptual framework in Figure 1). Three cases were purposively selected for additional member check (Supplementary File 2, member check 2), as detailed in Figure 2A-2C as representative examples.

For peer review only - http://bmjopen.bmj.com/site/about/guidelines.xhtml

Table 1. Parents' and children's characteristics (*n*=15)

Characteristic	Number
**Gender child, male** [a]	7
**Age child, years** [a]	
**4 -7**	5
**8 - 11**	8
**12 - 15**	1
**16 - 17**	1
**Urban area of residence** [a]	5
**Country of birth of both parents** [b]	
**The Netherlands**	15
**Educational level mother** [b, c]	
**Low**	1
**Intermediate**	6
**High**	8
**Educational level father** [b, c]	
**Low**	2
**Intermediate**	6
**High**	7
**Paid employment** [b]	
**Both parents**	14
**One parent**	1
**Marital status parents** [b]	
**Married / living together**	11
**Divorced**	4
**Present at interview** [b]	
**Child**	1
**Child and mother**	1
**Mother**	8
**Father**	2
**Both parents**	3
**Diagnosis after 6 months** [a]	
**Functional constipation**	9
**Irritable bowel syndrome**	2
**Functional constipation and functional abdominal pain**	2
**Functional abdominal pain**	2

[a] Clinician reported
[b] Patient reported
[c] Educational level was divided in low (primary and lower secondary education), intermediate (secondary (vocational) education) and high (Bachelor's degree or higher) (34,35).

For peer review only

Table 2. Subthemes per theme and factors positively and negatively influencing each theme

	Subtheme	*Positive influence*	*Negative influence*
Disease burden	**Duration**	Mild symptoms	Long duration of symptoms
		Stable symptoms	Recurring symptoms
		Short symptom duration	
	**Additional symptoms**		Additional symptoms (e.g. weight loss)
	**Impact**	No limitations due to symptoms	Symptoms impact rest of the family
		-	Child is limited in everyday life due to symptoms (e.g. school absenteeism, sports)
	**Concerns**	No concerns about symptoms	Concerns about symptoms
		No concerns because good explanation for symptoms (e.g. constipation, or runs in family)	Concerns about additional symptoms (e.g. weight loss)
		-	Concerns about impact on everyday life
	**History**	-	Parent recognise symptoms from their own medical history
		-	Other health care providers could not relieve the symptoms (e.g. psychotherapist, alternative medicine)
Doctor–patient relationship	**Pre-existing relationship**	Long-lasting doctor–patient relationship	First time at this GP
		Positive previous experience with GP	Negative previous experience with GP for chronic abdominal symptoms
	**Consultation**	Parents felt heard and understood	Not enough room in consultation for respondent's story
		GP focused on and comforted child	Short duration of consultation
		GP showed personal interest	GP did not answer main request
		GP fulfilled expectations	GP normalised or downplayed symptoms
		GP and respondents together decided treatment plan	GP showed no empathy
	**Follow-up**	GP planned follow-up consultation	No planned follow-up consultation
		Low threshold to make new GP appointment	Parent had to contact GP about continuation
		-	GP does not take enough initiative
		-	GP provided no help besides medical care (e.g. psychosocial care)
		-	GP did not provide a solution
Cognitive reassurance	**Explanation**	Matching explanatory model	Non-matching explanatory model
		Explanatory model fulfilled the respondent's expectation (e.g. constipation)	GP provided unclear or no explanatory model
		Respondents and GP together explored different explanatory models	GP did not seem to believe in explanatory model
		-	Respondents were unsure about explanation
	**Physical interventions**	Successful treatment (e.g. Forlax® relieved symptoms)	Unsuccessful treatment
		Physical examination confirmed diagnosis	Medical tests did not reveal an explanation
			Parents wished further medical investigation
		-	GP provided 'quick fix' for long-lasting problem (e.g. medication)

For peer review only - http://bmjopen.bmj.com/site/about/guidelines.xhtml

Disease burden

The disease burden experienced by parents influenced the expectations of GPs. Burden comprised the duration and severity of symptoms, the presence of additional symptoms (e.g., weight loss), the impact on everyday life, and the degree of concern about the symptoms (Table 2). Some parents experienced low disease burden (e.g., when in an early stage of symptoms), while others experienced chronic recurring GI symptoms that had limited everyday life for years.

> Mother (M8): *'For one year he was pain free and… during the last couple of months it returned. He has lost… weight, and the pain is limiting him in daily life… and in school. He arrived late to school because his belly hurt and he had to go to the toilet again. So, I just wanted further investigation.'*

Most parents with a low disease burden emphasised that they had no specific expectations of the GP.

> Mother (M6): *'Yes, exactly* [the GP fulfilled my expectations]. *Because it was not a severe situation, I did not have high expectations. I did not have a tense feeling,… so… GPs can help just by being friendly.'*

Parents had higher expectations with higher disease burdens, typically expressing concern that 'something must be wrong'. This often resulted in questions about the cause of symptoms and expectations for the GP to label symptoms, conduct further investigations, or refer to secondary care.

> Father (F1): '*At some point you are fed up… you want to know the answer, where it could come from? I know that my symptoms are clearly due to stress and everything, but I do not think that is the case for* [my daughter].*''*

> Mother (M3): *'And then I said 'You did not find anything but I would like it to be further investigated because 1) she is losing weight and 2) she just has an awful lot of pain attacks while turning pale, feeling bad, and not eating'. Her stool is almost never normal, so that cannot be right. But then, I got the response 'What investigations were you thinking of?'… I*

For peer review only - http://bmjopen.bmj.com/site/about/guidelines.xhtml

am no doctor! So… I said... 'Just send me to a paediatrician'… 'There must be a cause for this, this cannot be right'.'

Concern about symptoms varied among the parents. A couple of parents expressed concern over additional symptoms (e.g. fever), while others worried about the impact of symptoms on everyday life (e.g. school absenteeism and disturbed sleep) or the lack of explanation. By contrast, a few parents emphasised that they usually do not worry easily about their child's health and did not in this case.

Although both children mentioned that their symptoms affected them negatively during school and that they hoped the GP could find the cause and a solution, their parents had taken the initiative to consult the GP in both cases.

Doctor–patient relationship

The doctor–patient relationship comprised two elements: the pre-existing relationship (influencing both expectations and experiences) and the relationship during and after the consultation (influencing only experiences). Most parents reported a trusting relationship with their GP, with several having long-standing relationships and good previous experiences. One father expressed that his GP '*is not only a doctor, but above all, a good human being*'.

Parents emphasised the importance of feeling heard and understood by the GP during consultations. Additionally, most appreciated the GP comforting the child and directing his/her questions and explanations to the child.

> Mother (M4): '*If something is wrong… he just really takes the time for you. Sometimes I am in the waiting room and the previous consultation is running late. But, actually, I find that a good sign because it means he takes the time for you. Also, for children, he really takes the time.'*

Others felt their GP downplayed the child's symptoms.

> Mother (M8): *'But I do find that he could have shown a bit more empathy and… talked with my child a bit more. Because what he is feeling… is really what he is feeling. And now he has the sense that he was pretending. But it actually is… real. '*

For peer review only

Parents also felt comforted when the GP planned a follow-up consultation. This made them feel that the GP had acknowledged their concerns and the disease burden.

> Mother (M2): '*She completely examined* [my child], *we had a good conversation, and she* [said] *we will see and to call in 2 weeks to discuss it again… it was not like… manage it yourself and goodbye. Not at all.*'

Similarly, parents felt isolated when the GP did not plan a follow-up consultation.

> Mother (M1): '*If everything is ruled out* [and] *there are still symptoms, as a parent you want to do something… I would have appreciated a follow-up appointment, preferably already scheduled, so you can talk about it to find a good solution* together*. Although he cannot do anything with the test result* [everything is ruled out]*… let's keep talking…* like*... what can I help you with? Do you actually need help, or do you think you can handle it on your own?*'

When younger, the 17-year-old respondent mentioned that he found his GP intimidating. However, recent consultations for his chronic GI symptoms had changed this because the GP was considered '*friendly and neat*' towards him.

Reassurance

Parents expressed to feel reassured via two different pathways; cognitive and affective reassurance. Cognitive reassurance was achieved when the GP responded to or changed their beliefs about the symptoms (their cognitions). Whether the GP provided an explanatory model for the symptoms, and the choice of model, played prominent roles in achieving cognitive reassurance. Sometimes the GP gave an explanation that matched the parents' belief system (e.g. the GP confirmed their expectation that constipation caused the symptoms), making it relatively easy to establish cognitive reassurance. However, if their child's symptoms did not conform to the explanatory model, parents experienced insecurity about the explanation for the symptoms.

> Father (F5): '*That is the weird part because you know that the laxatives are for the constipation, but if a child suddenly has a fever and lies on the couch while having a hard time*

For peer review only - http://bmjopen.bmj.com/site/about/guidelines.xhtml

breathing… and has recovered within one hour… you do not think it is constipation… but… we did not know whether to call the GP again.'

How the GP conveyed the explanatory model also had a considerable impact on cognitive reassurance.

Child (Ch7): *'I don't know… it did feel like a bit of an oversimplification. Like… it felt like she did not know the answer herself…* [so] *she called it irritable bowel… that is how it felt.'*

This was especially the case for the two children, who both expressed that they did not understand the explanations because '*the GP talked too much*' and '*used too many difficult words*'.

Some parents emphasised that positive findings during physical examination or successful treatment made them believe in a certain explanatory model.

Mother (M4): *'Yes, I think* [the GP's final diagnosis was heartburn]*, although this was not said definitively…* [but] *since we put the bed up based on their advice,… I assume that it is related to heartburn.'*

Other parents expressed that they wanted the GP to perform more medical tests to find an explanatory model for the symptoms. However, none of the parents mentioned to feel reassured by a normal test result.

Not all parents wanted a specific explanatory model for the symptoms. Several stated that they just followed the GP's instructions (e.g. to take laxatives and return after 2 weeks with an update) and were comfortable that the GP had not provided a specific explanatory model early in the diagnostic process. In these cases, affective reassurance played a larger role, meaning parents felt reassured because the GP gave them the feeling there was no need to worry and they felt heard and understood by the GP.

Father (F14): *'She took both questions seriously… as well as our suggestions… and she found those suggestions plausible. So, she first performed a physical examination, looked at his tummy, knocked on it and she concluded that it was unlikely a purely physical cause.'*

For peer review only - http://bmjopen.bmj.com/site/about/guidelines.xhtml

Relationship between the themes and needs of parents and children

The three themes not only influenced the expectations and experiences of parents but also influenced each other (Figure 1), with this differing between interviews (see Figures 2A-2C). For example, the mother in Figure 2A suffered a high disease burden, which prompted her to seek an explanation (cognitive reassurance). In Figure 2B, the strong and long-term doctor–patient relationship made the father trust his GP when told that he should not worry about his son's symptoms (affective reassurance), without a need for an explanation for the symptoms or treatment (cognitive reassurance). By contrast, the doctor–patient relationship in Figure 2C was weak, resulting in a greater need for cognitive reassurance because the GP could not offer affective reassurance.

The relationship between the themes was also reinforced by whether the GP fulfilled or neglected the needs of parents. For example, in Figure 2B, the GP fulfilled the father's need for acknowledgement of the disease burden by planning a follow-up consultation. This, in turn, decreased the disease burden. Conversely, in Figure 2A, the GP downplayed the symptoms and disregarded the need for an explanation, increasing the disease burden and need for cognitive reassurance, respectively.

For peer review only

DISCUSSION

This qualitative interview study of the expectations and experiences when visiting a GP for chronic GI symptoms revealed three key themes: disease burden, GP–patient relationship, and reassurance. The basis of our conceptual framework is that the disease burden and pre-existing GP–patient relationship influence expectations. When a GP met parental expectations, a trusting doctor–patient relationship ensued, creating an environment that allowed affective and cognitive reassurance. Within this framework, specific needs of parents influenced the themes and their interrelationships: for the GP to acknowledge the disease burden, to search for an explanation together with the GP and to feel reassured. GPs should be aware that when they recognize and respond to these needs, they can positively influence the consultation experience of parents.

In this study, parents felt reassured through different pathways, with the success of a given reassurance strategy being situation specific. Sometimes a warm GP–patient relationship, instilling feelings of safety and comfort, was sufficient to provide reassurance (affective reassurance). Other times, parents needed cognitive reassurance, for example through an explanatory model for the symptoms. The need for an explanatory model is in concordance with a Norwegian study among parents of children with chronic abdominal pain (21). It is already known that specific concerns and beliefs about their symptoms (worrying cognitions) largely determine a patient's experience of cognitive reassurance (24). While some studies have argued that affective reassurance is a prerequisite to achieving cognitive reassurance (7), others state that affective reassurance only has a short-term and transient effect (9). Our findings suggest that any reassurance strategy is situation-specific, being related to both the GP–patient relationship and the disease burden.

Our findings fit with the theory of contextualising care, which advocates for medical decisions to be based on both biomedical evidence and the individual patient's context (36). This study found that important contextual factors, such as emotional state (e.g. parental concern) and the GP-patient relationship, affected parental expectations and experiences. Likewise, pre-existing beliefs about symptoms influenced parental adoption of the GP's explanatory model. Some

For peer review only - http://bmjopen.bmj.com/site/about/guidelines.xhtml

parents felt like their GP did not pay enough attention to contextual factors, resulting in 'contextual errors' (Figure 2A) (36), adding to the body of evidence that contextual factors should be used in medical decision making (36,37), as recommended by Dutch GP guidelines (38,39). Research has shown that this is especially important in patients with FSS (40). Since all children in our study were diagnosed with FGID, a symptom cluster of FSS, our study can be taken to endorse the use of contextual factors in these patients.

The Dutch GP guideline advises that practitioners schedule a follow-up consultation for children with chronic abdominal pain (38). Our findings support the importance of these follow-up consultations to parents and children, not only making them feel that the GP acknowledged their disease burden but also strengthening their relationship. Hence, a planned follow-up consultation is an important step in creating an enduring doctor–patient relationship, which is a core principle of general practice that has various positive effects on both patient health and health care costs (40–43).

To the best of our knowledge, there is no other study in which parents and children were interviewed about their expectations and experiences of a GP consultation when the child has chronic gastrointestinal symptoms. This is important because, in a gatekeeping system such as that in the Netherlands, the GP is the first physician to see a child with chronic GI symptoms and will typically manage most cases without referral to secondary care. Therefore, our results are transferable to other countries with gatekeeping systems, such as the United Kingdom and Canada (44). Additionally, the detailed description of our findings makes them transferable to the parents of children with other FSS (e.g. chronic fatigue) in general practice, because FSS share a similar underlying multifactorial pathophysiology (45).

This study knows some limitations. Despite the intention to interview children, only two children aged ≥12 years participated. While their expectations and experiences fit within the conceptual framework, they differed from the parent perspective. More importantly, the small number of participating children does not allow us to draw conclusions about their expectations and experiences. This implies that our findings are only transferable to parental expectations and

For peer review only - http://bmjopen.bmj.com/site/about/guidelines.xhtml

experiences. Future studies should explore children's perspectives, evaluate the framework's applicability to their expectations and experiences. Additionally, all participants had a typical Dutch cultural background. Almost a quarter of the Dutch population are first- or second generation immigrants, and each could have different expectations and experiences of health care that vary with their cultural beliefs (26,46,47). Although we purposively sampled mothers and fathers, we did not find any difference between them. The COVID-19 pandemic forced us to conduct video-interviews, which may have reduced credibility since in-person interviews are considered the 'gold-standard' for qualitative research. However, recent studies indicate that both methods produce similar data volumes, topic breadths, and personal information sharing (48,49). Additionally, video interviews are more popular among respondents due to scheduling ease, less intimate environment, and lack of travel (49).

A strength of this study is that all respondents were asked to provide feedback on a synopsis of their interview (member check 1) and the three respondents who constituted the representative cases were asked to provide more detailed feedback about our interpretation of their stories (member check 2). Another strength is that two researchers with different backgrounds performed all the interviews and main analyses, while another five researchers (two GPs, a primary care researcher, epidemiologist and a medical sociologist) participated in the coding, thereby offering different theory perspectives.

In conclusion, our findings suggest that an interrelationship exists between disease burden, the GP–patient relationship, and the reassurance given to parents and children who visit a GP for chronic GI symptoms. Although in our study the appropriate GP response was situation specific, planning a follow-up consultation is an important first step in addressing the needs of parents. We therefore recommend GPs to do accordingly, as it fosters an enduring GP–patient relationship, that is beneficial for all conditions, but particularly for patients with FSS. Further research should evaluate whether this framework also holds true for children. Additionally, it is important to explore the GP's views and experiences of these consultations. Finally, future studies should investigate whether using this framework in daily practice will help GPs manage children with

For peer review only - http://bmjopen.bmj.com/site/about/guidelines.xhtml

FGID and other FSS clusters and improve the quality of consultation experiences for parents and children.

For peer review only

For peer review only - http://bmjopen.bmj.com/site/about/guidelines.xhtml

Funding statement: This project was supported by ZonMW, Dutch Organization for Health Research and Development (project number 852001930).

Competing interests statement: None declared.

Authorship:

Sophie M. Ansems co-designed this study, recruited participants, performed all interviews, performed the initial analyses and interpretation of the data. She wrote the first and final draft of the manuscript.

Ilse N. Ganzevoort observed all interviews, co-performed the initial and final analysis and took part in the interpretation of the data. She revised the manuscript for important intellectual content.

Donald G. van Tol took part in the analysis and interpretation of the data. He revised the manuscript for important intellectual content.

Tryntsje Fokkema co-designed this study and took part in the analysis and interpretation of the data. She revised the manuscript for important intellectual content.

Marijke Olthof co-designed this study and took part in the interpretation of the data. She revised the manuscript for important intellectual content.

Marjolein Y. Berger co-designed this study and took part in the interpretation of the data. She revised the manuscript for important intellectual content.

Gea A. Holtman co-designed this study and took part in the interpretation of the data. She revised the manuscript for important intellectual content.

All authors approved the final version of this manuscript and agree to be accountable for all aspects of the work in ensuring that questions related to the accuracy or integrity of any part of the work are appropriately investigated and resolved.

Dr Robert Sykes (www.doctored.org.uk) provided editorial services.

For peer review only

Acknowledgements: The authors would like to thank all the respondents who volunteered and took part in this study.

Ethics approval statement: This study was approved by the Medical Research Ethics Committee of the University Medical Center Groningen (Research Register: 202000795).

Data sharing statement: Due to the confidential and sensitive nature of our data, data will not be made publicly available.

REFERENCES

1. Chitkara DK, Rawat DJ, Talley NJ. The Epidemiology of Childhood Recurrent Abdominal Pain in Western Countries: A Systematic Review. Am J Gastroenterol. 2005 Aug;100(8):1868–75.

2. Gieteling MJ, Lisman-van Leeuwen Y, van der Wouden JC, Schellevis FG, Berger MY. Childhood nonspecific abdominal pain in family practice: incidence, associated factors, and management. Ann Fam Med. 2011;9(4):337–43.

3. Spee LAA, Lisman-Van Leeuwen Y, Benninga MA, Bierma-Zeinstra SMA, Berger MY. Prevalence, characteristics, and management of childhood functional abdominal pain in general practice. Scand J Prim Health Care. 2013 Dec [;31(4):197–202.

4. Hyams JS, Di Lorenzo C, Saps M, Shulman RJ, Staiano A, van Tilburg M. Childhood Functional Gastrointestinal Disorders: Child/Adolescent. Gastroenterology. 2016 May 1;150(6):1456-1468.e2.

5. Bonvanie IJ, Kallesøe KH, Janssens KAM, Schröder A, Rosmalen JGM, Rask CU. Psychological Interventions for Children with Functional Somatic Symptoms: A Systematic Review and Meta-Analysis. J Pediatr. 2017 Aug 1;187:272-281.e17.

6. Olde Hartman TC, Hassink-Franke LJ, Lucassen PL, Van Spaendonck KP, Van Weel C. Explanation and relations. How do general practitioners deal with patients with persistent medically unexplained symptoms: A focus group study. BMC Fam Pract. 2009;10:68.

7. Giroldi E, Veldhuijzen W, Leijten C, Welter D, Weijden T van der, Muris J, et al. 'No need to

For peer review only - http://bmjopen.bmj.com/site/about/guidelines.xhtml

worry': an exploration of general practitioners' reassuring strategies. BMC Fam Pract. 2014 Jul 7;15(1):133.

8. Terpstra T, Gol JM, Lucassen PLBJ, Houwen J, van Dulmen S, Berger MY, et al. Explanations for medically unexplained symptoms: a qualitative study on GPs in daily practice consultations. Fam Pract. 2019 Aug 8;37(1):124–30.

9. Pincus T, Holt N, Vogel S, Underwood M, Savage R, Walsh DA, et al. Cognitive and affective reassurance and patient outcomes in primary care: A systematic review. Pain. 2013;154(11):2407–16.

10. Brodwall A, Brekke M. General practitioners' experiences with children and adolescents with functional gastro-intestinal disorders: a qualitative study in Norway. Scand J Prim Health Care. 2021;39(4):543–51.

11. Petrie KJ, Müller JT, Schirmbeck F, Donkin L, Broadbent E, Ellis CJ, et al. Effect of providing information about normal test results on patients' reassurance: Randomised controlled trial. Br Med J. 2007 Feb 17;334(7589):352–4.

12. McDonald I, Daly J, Jelinek V, Panetta F, Gutman J. Opening Pandora's box: the unpredictability of reassurance by a normal test result. BMJ. 1996;313(7053):329–32.

13. Ring A, Dowrick CF, Humphris GM, Davies J, Salmon P. The somatising effect of clinical consultation: What patients and doctors say and do not say when patients present medically unexplained physical symptoms. In: Social Science and Medicine. Pergamon; 2005. p. 1505–15.

14. Lisman-van Leeuwen Y, Spee LAA, Benninga MA, Bierma-Zeinstra SMA, Berger MY. Prognosis of abdominal pain in children in primary care-A prospective cohort study. Ann Fam Med. 2013;11(3):238–44.

15. Scharff L. Recurrent abdominal pain in children: a review of psychological factors and treatment. Clin Psychol Rev. 1997;17(2):145–66.

16. Ayonrinde OT, Ayonrinde OA, Adams LA, Sanfilippo FM, O' Sullivan TA, Robinson M, et al. The relationship between abdominal pain and emotional wellbeing in children and

For peer review only

For peer review only - http://bmjopen.bmj.com/site/about/guidelines.xhtml

adolescents in the Raine Study. Sci Reports 2020 101. 2020 Feb 3;10(1):1–11.

17. Brodwall A, Brekke M. Original research: Children's and adolescents' experiences with functional abdominal pain: a qualitative study in Norway. BMJ Open. 2021 Sep 13;11(9):48949.

18. Cole MAT, Qu'd D, Wild MG, Russell AC, Caillet AR, Stone AL. "My Body Hates Me": A Qualitative Analysis of the Experience of Functional Nausea in Adolescent Girls and Their Mothers. Children. 2020 Aug 1;7(8).

19. Fortin S, Gauthier A, Gomez L, Faure C, Bibeau G, Rasquin A. Uncertainty, culture and pathways to care in paediatric functional gastrointestinal disorders. http://dx.doi.org/101080/136484702013853026. 2013 Dec 1;20(3):311–23.

20. Smart S, Cottrell D. Going to the doctors: the views of mothers of children with recurrent abdominal pain. Child Care Health Dev. 2005 May 1;31(3):265–73.

21. Brodwall A, Glavin K, Lagerløv P. Parents' experience when their child has chronic abdominal pain: a qualitative study in Norway. BMJ Open. 2018;8:21066.

22. Brekke M, Brodwall A. Understanding parents' experiences of disease course and influencing factors: A 3-year follow-up qualitative study among parents of children with functional abdominal pain. BMJ Open. 2020 Aug 30;10(8).

23. van Tilburg MAL, Venepalli N, Ulshen M, Freeman KL, Levy R, Whitehead WE. Parents' worries about recurrent abdominal pain in children. Gastroenterol Nurs. 2006 Jan 1;29(1):50–5; quiz 56–7.

24. Giroldi E, Veldhuijzen W, Mannaerts A, Weijden T van der, Bareman F, Vleuten C van der. "Doctor, please tell me it's nothing serious": an exploration of patients' worrying and reassuring cognitions using stimulated recall interviews. BMC Fam Pract. 2014 Apr 23;15(1):73.

25. Cole MAT, Qu'd D, Wild MG, Russell AC, Caillet AR, Stone AL. "My Body Hates Me": A Qualitative Analysis of the Experience of Functional Nausea in Adolescent Girls and Their Mothers. Children. 2020 Aug;7(8).

For peer review only - http://bmjopen.bmj.com/site/about/guidelines.xhtml

26. Fortin S, Gauthier A, Gomez L, Faure C, Bibeau G, Rasquin A. Uncertainty, culture and pathways to care in paediatric functional gastrointestinal disorders. http://dx.doi.org/101080/136484702013853026. 2013 Dec;20(3):311–23.

27. Hennink M, Hutter I, Bailey A. Qualitative Research Methods. SAGE Publications Sage CA: Los Angeles, CA; 2020. Chapter 3: The Design Cycle.

28. Tong A, Sainsbury P, Craig J. Consolidated criteria for reporting qualitative research (COREQ): a 32-item checklist for interviews and focus groups. Int J Qual Heal Care. 2007 Dec 1;19(6):349–57.

29. O'Brien BC, Harris IB, Beckman TJ, Reed DA, Cook DA. Standards for reporting qualitative research: a synthesis of recommendations. Acad Med. 2014;89(9):1245–51.

30. Ansems S, Berger M, van Rheenen PF, Vermeulen K, Beugel G, Couwenberg M, et al. Effect of faecal calprotectin testing on referrals for children with chronic gastrointestinal symptoms in primary care: study protocol for a cluster randomised controlled trial. BMJ Open. 2021 Jul 23;11(7):e045444.

31. Collins CS, Stockton C. The Theater of Qualitative Research: The Role of the Researcher/Actor. Int J Qual Methods. 2022 May 1;21. A

32. Hennink M, Hutter I, Bailey A. Qualitative Research Methods. 2020. Chapter 7: In-depth interviews (page 127).

33. Braun V, Clarke V. Thematic Analysis. In: APA Handbook of Research Methods in Psychology: Vol 2 Resarch Designs. 2012. p. 57–71.

34. UNESCO Institute for Statistics. International Standard Classification of education - ISCED. 2011. http://uis.unesco.org/sites/default/files/documents/international-standard-classification-of-education-isced-2011-en.pdf

35. Centraal Bureau voor Statistiek (CBS). Niveau ISCED 2011. https://www.cbs.nl/nl-nl/onze-diensten/methoden/classificaties/onderwijs-en-beroepen/isced/niveau-isced-2011

36. Weiner SJ, Schwartz A. Contextual errors in medical decision making: Overlooked and

For peer review only

understudied. Acad Med. 2016 May 1;91(5):657–62.

37. Weiner SJ, Schwartz A, Weaver F, Goldberg J, Yudkowsky R, Sharma G, et al. Contextual errors and failures in individualizing patient care: A multicenter study. Ann Intern Med. 2010 Jul 20;153(2):69–75.

38. Gieteling MJ, van Dijk PA, de Jonge AH, Albeda FW, Berger MY, Burgers JS, et al. NHG-Standaard Buikpijn bij kinderen. Huisarts Wet. 2012;55(9):404–9.

39. Olde Hartman TC, Blankenstein AH, Molenaar B et al. NSS. NHG Guideline on medically unexplained symptoms (MUS). Huisarts Wet. 2013;56(5):222–30.

40. Kornelsen J, Atkins C, Brownell K, Woollard R. The Meaning of Patient Experiences of Medically Unexplained Physical Symptoms: http://dx.doi.org/101177/1049732314566326. 2015 Jan 12;26(3):367–76. A

41. Saultz JW, Lochner J. Interpersonal continuity of care and care outcomes: a critical review. Ann Fam Med. 2005 Mar;3(2):159–66.

42. Haggerty JL, Reid RJ, Freeman GK, Starfield BH, Adair CE, McKendry R. Continuity of care: a multidisciplinary review. BMJ . 2003 Nov 20;327(7425):1219–21.

43. Skarshaug LJ, Kaspersen SL, Bjørngaard JH, Pape K. Original research: How does general practitioner discontinuity affect healthcare utilisation? An observational cohort study of 2.4 million Norwegians 2007–2017. BMJ Open. 2021 Feb 16;11(2):e042391.

44. Thomson S, Osborn R, Squires D, Reed S. International Profiles of Health Care Systems: Australia, Canada, Denmark, England, France, Germany, Italy, Japan, the Netherlands, New Zealand, Norway, Sweden, Switzerland, and the United States | Commonwealth Fund . 2011.

45. Fink P, Schröder A. One single diagnosis, bodily distress syndrome, succeeded to capture 10 diagnostic categories of functional somatic syndromes and somatoform disorders. J Psychosom Res. 2010 May 1;68(5):415–26.

46. Hoeveel mensen met een migratieachtergrond wonen in Nederland? https://www.cbs.nl/nl-nl/dossier/dossier-asiel-migratie-en-integratie/hoeveel-mensen-

For peer review only - http://bmjopen.bmj.com/site/about/guidelines.xhtml

met-een-migratieachtergrond-wonen-in-nederland-

47. Nijman RG, Oostenbrink R, Dons EM, Bouwhuis CB, Moll HA. Parental fever attitude and management: Influence of parental ethnicity and child's age. Pediatr Emerg Care. 2010 May;26(5):339–42.

48. Krouwel M, Jolly K, Greenfield S. Comparing Skype (video calling) and in-person qualitative interview modes in a study of people with irritable bowel syndrome – an exploratory comparative analysis. BMC Med Res Methodol. 2019 Nov 29;19(1).

49. Jenner BM, Myers KC. Intimacy, rapport, and exceptional disclosure: a comparison of in-person and mediated interview contexts. Int J Soc Res Methodol. 2019 Mar 4;22(2):165–77.

Figure Legends.

Figure 1. Conceptual framework. All 3 themes (blue rectangles) influenced the expectations and experiences of parents, and their experiences were influenced by whether the GP met their expectations. GPs can influence the relationships between themes by responding to the needs of parents and children (displayed in bold, next to the arrows).

Figure 2A. Conceptual framework exemplified by Case 8. All themes are shown in red, indicating that these were negatively influenced, because the GP did not respond to the mother's needs. The 3 bold arrows indicate a strong relationship between the themes.

Figure 2B. Conceptual framework exemplified by Case 12. The themes shown in green were positively influenced because the GP responded to the father's needs. The theme shown in grey (cognitive reassurance) was not prominent during the interview. Bold arrows indicate the strong relationship between themes.

Figure 2C. Conceptual framework exemplified by Case 9. The red theme was negatively influenced because the GP did not respond to mother's needs. The grey themes were not prominent during the interview. The bold arrows indicate strong relationships between themes, and the grey arrow indicates that this relationship was not prominent during the interview.

For peer review only - http://bmjopen.bmj.com/site/about/guidelines.xhtml

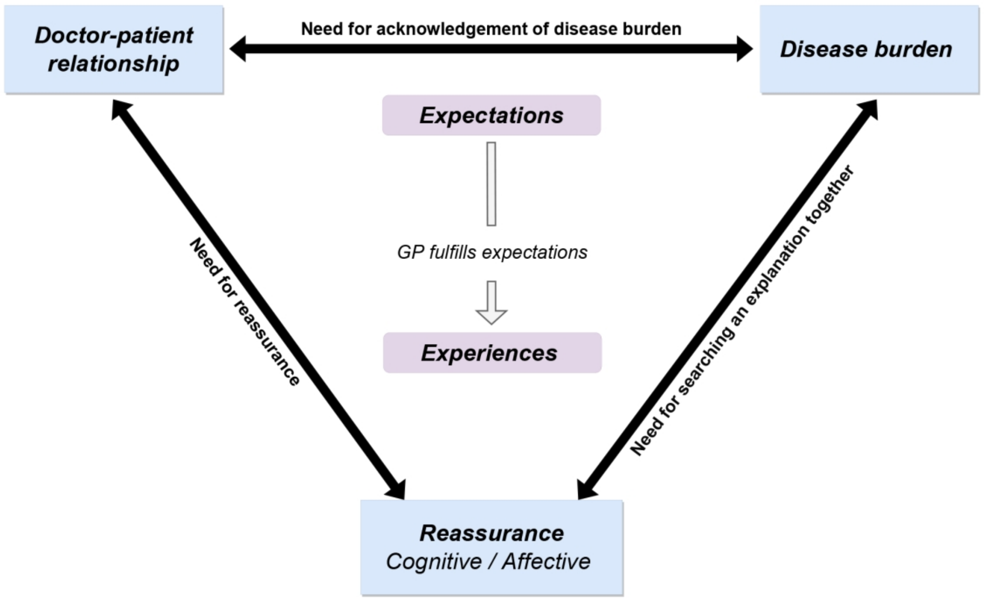

Figure 1. All 3 themes (blue rectangles) influenced the expectations and experiences of parents, and their experiences were influenced by whether the GP met their expectations. GPs can influence the relationships between themes by responding to the needs of parents and children (displayed in bold, next to the arrows).

41x24mm (1200 x 1200 DPI)

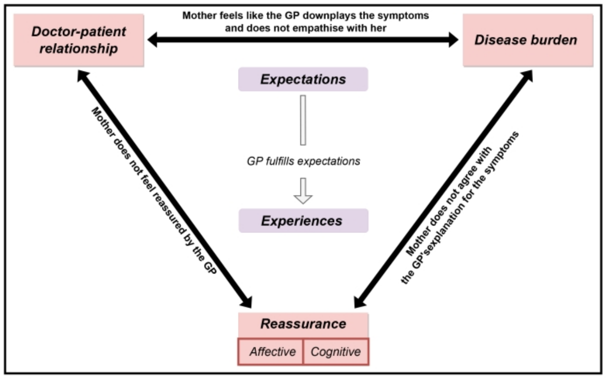

Figure 2A

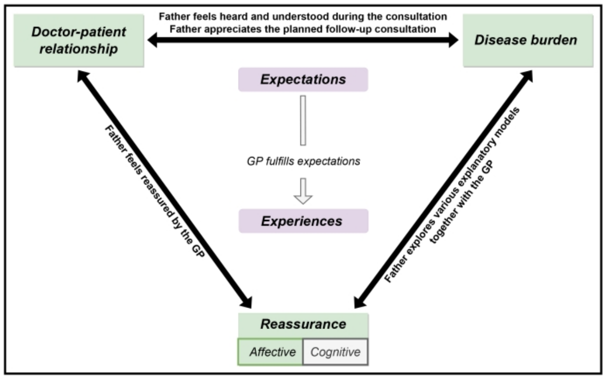

Figure 2B

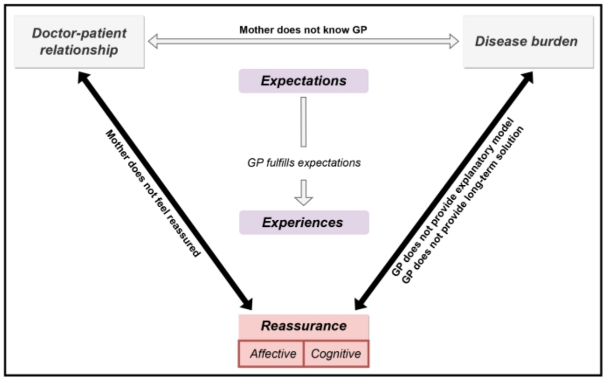

Figure 2C

Figure 2A. Conceptual framework exemplified by Case 8. All themes are shown in red, indicating that these were negatively influenced, because the GP did not respond to the mother's needs. The 3 bold arrows indicate a strong relationship between the themes.
Figure 2B. Conceptual framework exemplified by Case 12. The themes shown in green were positively influenced because the GP responded to the father's needs. The theme shown in grey (cognitive reassurance) was not prominent during the interview. Bold arrows indicate the strong relationship between themes.
Figure 2C. Conceptual framework exemplified by Case 9. The red theme was negatively influenced because the GP did not respond to mother's needs. The grey themes were not prominent during the interview. The bold arrows indicate strong relationships between themes, and the grey arrow indicates that this relationship was not prominent during the interview.

52x103mm (1200 x 1200 DPI)

Supplementary File 1. Interview guide [a]

I. Introduction

- Formal introduction and explantation about the interview, including the following:
  - Study aim, "to evaluate the expectations and experiences of children with chronic gastrointestinal symptoms and their parents when visiting their GP." Explain that our goal is to improve the care provided to these children.
  - That interviews will be audio- and video-recorded for processing and analysis.
  - That all respondents will be offered a final check to indicate if they agree.
- Ask if they have any last questions or remarks before recording starts.

START RECORDING

- Explain the following:
  - That shared information will be handled confidentially and anonymously and will not be shared with the relevant GP.
  - Duration: maximum of 1 hour.
  - A synopsis of interview will be sent for a member check after the interview.

II. Opening question

Could you tell me something about your/your child's gastrointestinal symptoms?

III. Topics

1. Incentives

Why did you decide to visit your GP for the gastrointestinal symptoms?

- Expansion questions:
  - Were you concerned about the symptoms? If so, what was your biggest concern? (Serious illness / impact on daily life / school absenteeism / child's suffering.)
  - What are your views about the cause of the symptoms (serious illness / stress related / innocent)?
  - How would you describe your relationship with your GP?

2. Expectations

I would like you to go back to the day you visited the GP for the first time for the gastrointestinal symptoms. What did you hope what was going to happen?

- Possible expectations:
  - Sympathetic ear.
  - Rule out physical illness (further medical investigations).
  - Find an explanation for the symptoms.

[a] This guide has been altered slightly to make it comprehensible outside the research group

For peer review only

- Symptom relief (e.g. a change in eating pattern or medication).
- Advice on how to deal with the symptoms (whether to go to school / whether to give the child attention).

3. Experiences

How did you feel when you left the GP practice?

- Suggested feelings:
  - Taken seriously / not understood / like I waisted the GP's time / relieved / concerned/ anxious
- Expansion questions
  - What made you feel like that?
  - How did the GP contribute to this feeling?

Did the GP fulfil your expectations?

How would you score the GP's performance in the consultation on a scale from 1 to 10?

- Expansion questions: (e.g., if the answer is 7/10) what should the GP have done to turn the 7 into an 8?
- Possible improvements: Further medical investigations / referral / treatment / listen better / better explanation about the cause of the symptoms.

If you could ask the GP a question about the gastrointestinal symptoms right now, what would it be?

- Notes: Diagnosis / further steps in treatment / guidance over time

Do you now hold different views about the symptoms compared to before the GP consultation?

4. Explanation for the symptoms

What explanation did the GP provide for the symptoms? Did this explanation help you?

IV. Finishing question

Before I end the recording: is there anything else you would like to share with me?

END RECORDING

V. Important questions for all themes:

If I would ask your parents the same question, what would they answer?

If I would ask your son/daughter the same question, what would he/she answer?

[a] This guide has been altered slightly to make it comprehensible outside the research group

For peer review only

For peer review only

Supplementary File 2. Responses to the member check

Member check 1: Synopsis of the interview – All 15 respondents

Four respondents informed us that they agreed with the synopsis.

Ten respondents did not respond to the synopsis.

One mother made amendments to the synopsis clarifying her daughter's specific symptoms, why she had decided to visit the GP and how she and her husband deal with problems concerning the health of their children.

Member check 2. Conceptual framework exemplified by the interview – 3 respondents

One respondent informed us that they agreed with how their story was represented in the conceptual framework.

One respondent did not respond to our e-mail.

One respondent let us know the following via e-mail:

> *The way you have outlined the story is correct for 90%. The last part, about the relationship between me and the GP, is not completely right. When I went to the doctor for my daughter, we were seen by a representative of the GP practice. So not my own doctor. I assume her work is monitored, since my own GP called me to ask if I wanted to participate in the trial. This was in response to my visit. So, he did receive the signal. However, it is true that he also did not perform further investigations to find the cause of my daughter's symptoms. However, I do completely trust my own GP. I do not visit him often, but when I visit him, I feel taken seriously. For example, my other daughter has been admitted to the hospital due to the RS virus because he took her symptoms seriously. But this time it was a representative. Maybe it is true that my trust in her is not as high as in my own GP. But I thought that the last part in the text somewhat wronged my own GP. So that is my only remark. Besides that: fine!*

After making changes in the document clarifying that the GP discussed was not the mother's regular GP, she responded as follows:

> *The adjustment is very minimal. The reader could still interpret the story differently (as seen from my perspective). However, I do understand that this text is created with a certain aim. From that perspective, very well, use it like this. Thank you for discussing this with me.*

For peer review only

Supplementary File 2. Standards for Reporting Qualitative Research (SRQR) checklist

Standards for Reporting Qualitative Research (SRQR)*

http://www.equator-network.org/reporting-guidelines/srqr/

Title and abstract

	**Page/line no(s).**
**Title** - Concise description of the nature and topic of the study Identifying the study as qualitative or indicating the approach (e.g., ethnography, grounded theory) or data collection methods (e.g., interview, focus group) is recommended	Page 1
**Abstract** - Summary of key elements of the study using the abstract format of the intended publication; typically includes background, purpose, methods, results, and conclusions	Page 2

Introduction

**Problem formulation** - Description and significance of the problem/phenomenon studied; review of relevant theory and empirical work; problem statement	Page 5
**Purpose or research question** - Purpose of the study and specific objectives or questions	Page 5+6

Methods

**Qualitative approach and research paradigm** - Qualitative approach (e.g., ethnography, grounded theory, case study, phenomenology, narrative research) and guiding theory if appropriate; identifying the research paradigm (e.g., postpositivist, constructivist/ interpretivist) is also recommended; rationale**	Page 7 Line 3-4
**Researcher characteristics and reflexivity** - Researchers' characteristics that may influence the research, including personal attributes, qualifications/experience, relationship with participants, assumptions, and/or presuppositions; potential or actual interaction between researchers' characteristics and the research questions, approach, methods, results, and/or transferability	Page 8 Line 6-13
**Context** - Setting/site and salient contextual factors; rationale**	Page 7 Line 4-6
**Sampling strategy** - How and why research participants, documents, or events were selected; criteria for deciding when no further sampling was necessary (e.g., sampling saturation); rationale**	Page 7 Line 12-22
**Ethical issues pertaining to human subjects** - Documentation of approval by an appropriate ethics review board and participant consent, or explanation for lack thereof; other confidentiality and data security issues	Page 7 Line 22-25<br>Page 7 Line 9-10

For peer review only - http://bmjopen.bmj.com/site/about/guidelines.xhtml

**Data collection methods** - Types of data collected; details of data collection procedures including (as appropriate) start and stop dates of data collection and analysis, iterative process, triangulation of sources/methods, and modification of procedures in response to evolving study findings; rationale**	Page 7+8
**Data collection instruments and technologies** - Description of instruments (e.g., interview guides, questionnaires) and devices (e.g., audio recorders) used for data collection; if/how the instrument(s) changed over the course of the study	Page 7+8<br>Supp. File 1
**Units of study** - Number and relevant characteristics of participants, documents, or events included in the study; level of participation (could be reported in results)	Table 1<br>Page 10 3-9
**Data processing** - Methods for processing data prior to and during analysis, including transcription, data entry, data management and security, verification of data integrity, data coding, and anonymization/de-identification of excerpts	Page 7-9
**Data analysis** - Process by which inferences, themes, etc., were identified and developed, including the researchers involved in data analysis; usually references a specific paradigm or approach; rationale**	Page 8-9
**Techniques to enhance trustworthiness** - Techniques to enhance trustworthiness and credibility of data analysis (e.g., member checking, audit trail, triangulation); rationale**	Page 8 Line 19-20<br>Page 9 Line 6-8

Results/findings

**Synthesis and interpretation** - Main findings (e.g., interpretations, inferences, and themes); might include development of a theory or model, or integration with prior research or theory	Page 10-17
**Links to empirical data** - Evidence (e.g., quotes, field notes, text excerpts, photographs) to substantiate analytic findings	Page 10-17

Discussion

**Integration with prior work, implications, transferability, and contribution(s) to the field** - Short summary of main findings; explanation of how findings and conclusions connect to, support, elaborate on, or challenge conclusions of earlier scholarship; discussion of scope of application/generalizability; identification of unique contribution(s) to scholarship in a discipline or field	Page 18-21
**Limitations** - Trustworthiness and limitations of findings	Page 18-19

Other

**Conflicts of interest** - Potential sources of influence or perceived influence on study conduct and conclusions; how these were managed	Page 22
**Funding** - Sources of funding and other support; role of funders in data collection, interpretation, and reporting	Page 22

For peer review only - http://bmjopen.bmj.com/site/about/guidelines.xhtml

*The authors created the SRQR by searching the literature to identify guidelines, reporting standards, and critical appraisal criteria for qualitative research; reviewing the reference lists of retrieved sources; and contacting experts to gain feedback. The SRQR aims to improve the transparency of all aspects of qualitative research by providing clear standards for reporting qualitative research.

**The rationale should briefly discuss the justification for choosing that theory, approach, method, or technique rather than other options available, the assumptions and limitations implicit in those choices, and how those choices influence study conclusions and transferability. As appropriate, the rationale for several items might be discussed together.

Reference:

O'Brien BC, Harris IB, Beckman TJ, Reed DA, Cook DA. **Standards for reporting qualitative research: a synthesis of recommendations.** *Academic Medicine*, Vol. 89, No. 9 / Sept 2014
DOI: 10.1097/ACM.0000000000000388

For peer review only

For peer review only - http://bmjopen.bmj.com/site/about/guidelines.xhtml

SUPPLEMENTAL FILE S1: COREQ 32-ITEM CHECKLIST

Tong A, Sainsbury P, Craig J. (2007) Consolidated criteria for reporting qualitative research (COREQ): a 32-item checklist for interviews and focus groups. International Journal for Quality in Healthcare: 19:349 – 357

No. Item	Guide questions/description	Reported on Page #
Domain 1: Research team and reflexivity		
1. Inter viewer/facilitator	Which author/s conducted the interview?	8
2. Credentials	What were the researcher's credentials?	8
3. Occupation	What was their occupation at the time of the study?	8
4. Gender	Was the researcher male or female?	8
5. Experience and training	What experience or training did the researcher have?	8
6. Relationship with participants established	Was a relationship established prior to study commencement?	10
7. Participant knowledge of the interviewer	What did the participants know about the researcher?	8
8. Interviewer characteristics	What characteristics were reported about the inter viewer/facilitator?	8
Domain 2: study design		
9. Methodological orientation and Theory	What methodological orientation was stated to underpin the study?	7
10. Sampling	How were participants selected?	7
11. Method of approach	How were participants approached?	7+10
12. Sample size	How many participants were in the study?	10
13. Non-participation	How many people refused to participate or dropped out? Reasons?	10
14. Setting of data collection	Where was the data collected?	7
15. Presence of non-participants	Was anyone else present besides the participants and researchers?	8
16. Description of sample	What are the important characteristics of the sample?	Table 1
17. Interview guide	Were questions, prompts, guides provided by the authors?	Suppl. file 1
18. Repeat interviews	Were repeat interviews carried out?	NA
19. Audio/visual recording	Did the research use audio or visual recording to collect the data?	8
20. Field notes	Were field notes made during and/or	9

For peer review only

	after the inter view?	
21. Duration	What was the duration of the interviews	8
22. Data saturation	Was data saturation discussed?	7+10
23. Transcripts returned	Were transcripts returned to participants for comment and/or correction?	8+9<br>Suppl File 2
Domain 3: analysis and findings		
24. Number of data coders	How many data coders coded the data?	8+9
25. Description of the coding tree	Did authors provide a description of the coding tree?	NA
26. Derivation of themes	Were themes identified in advance or derived from the data?	7
27. Software	What software, if applicable, was used to manage the data?	9
28. Participant checking	Did participants provide feedback on the findings?	Page 10<br>Suppl File 2
29. Quotations presented	Were participant quotations presented to illustrate the themes/findings? Was each quotation identified?	13-16
30. Data and findings consistent	Was there consistency between the data presented and the findings?	10-17
31. Clarity of major themes	Were major themes clearly presented in the findings?	13-16
32. Clarity of minor themes	Is there a description of diverse cases or discussion of minor themes?	13-16

For peer review only
